# Innovative Self‐Assembly of 15‐Mer Chimeric α‐Peptide–Oligourea Foldamers toward Cl^−^‐Selective Ion Channels

**DOI:** 10.1002/smsc.202300352

**Published:** 2024-05-30

**Authors:** Chiranjit Dutta, Pannaga Krishnamurthy, Dandan Su, Jianwei Li, Sung Hyun Yoo, Gavin W. Collie, Morgane Pasco, Jingsong Fan, Min Luo, Mihail Barboiu, Gilles Guichard, R. Manjunatha Kini, Prakash Kumar

**Affiliations:** ^1^ Department of Biological Sciences National University of Singapore Singapore 117543 Singapore; ^2^ NUS Environmental Research Institute (NERI) National University of Singapore Singapore 117411 Singapore; ^3^ Institut Européen des Membranes Adaptive Supramolecular Nanosystems Group University of Montpellier ENSCM, CNRS Place Eugène Bataillon, CC 047 F‐34095 Montpellier France; ^4^ Institut Européen de Chimie et Biologie Univ. Bordeaux, CNRS, Bordeaux INP, CBMN, UMR 5248 2 rue Robert Escarpit F‐33600 Pessac France; ^5^ Discovery Sciences R&D, AstraZeneca Cambridge UK; ^6^ Department of Pharmacology Yong Loo Lin School of Medicine National University of Singapore Singapore 117559 Singapore

**Keywords:** foldamers, helices, ion channels, lipid membranes, self‐assembly, structure

## Abstract

Constructing artificial ion channels is a challenging task. Herein, the de novo design of transmembrane ion channels made up of amphiphilic peptide–oligourea chimeric helices is described. They consist of an oligourea segment (7‐mer) attached to the C‐terminus of a short peptide (8‐mer). Mass spectrometry (MS) and transmission electron microscopy (TEM) analyses show that in an aqueous solution, two of these chimeras (HPU‐E and HPU‐N) independently form defined oligomeric structures. TEM also shows that they form fiber bundles. The third related chimera HPU‐F does not oligomerize (MS) but forms spherical nanostructures (TEM). HPU‐E and HPU‐N exhibit anion transport activity across lipid bilayers via antiport mechanism (HPU‐N > HPU‐E). The anion selectivity of HPU‐N is Cl^−^>NO_3_^−^ > Br^−^>SCN^−^ > I^−^ > AcO^−^>F^−^, which can be due to anion binding within the channels rather than size exclusion. Patch‐clamp data support HPU‐N's Cl^−^ selectivity (PCl^−^/PI^−^ = 3.26). X‐ray crystal structure (1.77 Å) of HPU‐N reveals well‐packed α‐helices, and cryo‐electron microscopy data shows the formation of nanotubes (13.7 Å diameter pores) and transmembrane channels. The study shows that α‐peptide–oligourea‐based de novo design can yield unique bioactive molecules with defined structures and functions.

## Graphical abstract

Chimeric foldamers synthesized by linking an *α*‐peptide to the N‐terminus of an oligourea possess distinct hydrophobic and hydrophilic faces. Two such foldamers exhibit fiber‐like self‐assembly and demonstrate high anion transport activity in lipid bilayers. The quaternary structure from X‐ray crystallography revealed helical bundles and hydrophilic pores of ≈20 Å diameter. Cryo‐EM of liposome‐embedded chimera demonstrated self‐assembled channel‐like structures.

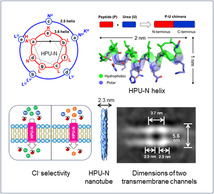

## 1 Introduction

Transmembrane ion transport is involved in various physiological processes that are controlled by specialized transporters or ion channel proteins.^[^ ^1^, ^2^ ^]^ Alteration in ion transport leads to several diseases that are collectively called channelopathies.^[^ ^3^ ^]^ Transporters and channels transport ions and molecules across membranes through thermodynamically different mechanisms.^[^ ^4^ ^]^ While transporters employ a binding energy‐dependent process, ions passively diffuse across ion channels. The chloride channels, belonging to the anion transporter family, conduct chloride ions across the membrane and modulate key cellular processes including cell volume regulation, muscle contraction, neuroexcitation, and endosomal and lysosomal acidification.^[^ ^1^, ^3^ ^]^ As opposed to cation channels that generally show selectivity for a single cation, chloride channels transport chloride and other anions such as halides, pseudohalide (SCN^−^), and bicarbonate.^[^ ^1^, ^3^ ^]^ Mutations in chloride channels cause genetic diseases, with the most prevalent illness being cystic fibrosis.^[^ ^3^ ^]^ Tremendous efforts have been made to develop new drug molecules to restore the normal ion channel function in diseased cells.^[^ ^3^, ^5^ ^]^

Concurrently, insights from basic research have stimulated the design of many synthetic molecular systems that mimic structures and functions of native ion channels for various biotechnological applications.^[^ ^6^, ^7^ ^]^ Although challenging, de novo peptide design and engineering offer exciting prospects to create sequences that self‐assemble into membranes to form ion channels.^[^ ^8^, ^9^, ^10^, ^11^ ^]^ One promising approach is that recently developed by Woolfson and co‐workers,^[^ ^11^ ^]^ which uses α‐helical peptides with the ability to self‐assemble into water‐soluble α‐helical barrels (αHBs) as a starting point. αHBs are unique coiled‐coil structures composed of 5–9 helices with a narrow central channel ranging in diameters from 9.5 to 11.5 Å.^[^ ^11^, ^12^ ^]^ Water‐soluble αHBs were converted into membrane‐spanning cation‐selective ion channels by further engineering the sequence, first by creating a polar interior and then by adding hydrophobic residues projecting outward to facilitate membrane insertion.^[^ ^11^ ^]^

Alternatively, the ability to create non‐natural sequence‐defined synthetic oligomers with high folding propensity (i.e., foldamers^[^ ^13^, ^14^ ^]^) suggests that self‐assembly principles at work for α‐helical peptides may also be used to construct transmembrane channels from helical foldamers.^[^ ^15^, ^16^, ^17^, ^18^, ^19^, ^20^ ^]^ Compared to natural α‐peptides, non‐natural backbones may provide increased resistance to proteolysis, high secondary structure predictability, and sequence diversity. However, the creation of precise, bioinspired nanostructures by self‐assembly of foldamers in aqueous solution remains challenging and examples are limited.^[^ ^13^, ^16^, ^17^, ^18^, ^20^ ^]^ In our group, we have introduced chiral aliphatic N,N’‐linked urea oligomers (i.e., oligoureas) as peptidomimetic foldamers without amino acids and have shown that amphiphilic water‐soluble oligourea sequences can be used to construct nanometer‐scale assemblies mimicking protein quaternary structures. In particular, the assembly process can be oriented toward compact (i.e., helix bundles) or extended nanostructures (i.e., superhelical hydrophilic channels) in aqueous conditions by de novo sequence manipulation.^[^ ^21^, ^22^ ^]^ We recently found that oligoureas with the propensity to form nanotubes with a polar interior in aqueous solution can insert into membranes to generate artificial water channels.^[^ ^23^ ^]^ A remarkable feature of oligoureas is that they can be interfaced with α‐helical peptides to generate hybrid oligomers with the propensity to form new helical structures in which the 2.5 helical structure of the oligourea segment and the α‐peptide helix are connected by a regular intramolecular network of H bonds.^[^ ^24^, ^25^, ^26^ ^]^ The ion transport potential of such peptide–oligourea chimeric foldamers is not known. We hypothesized that these hybrid sequences may exhibit novel self‐assembled structures not observed in either canonical peptides or pure oligourea foldamers. Highly selective recognition in transmembrane channels is a significant challenge, and utilizing mixed arrangements of residues in the peptide–oligourea helix, along with unique self‐assembly, could facilitate the achievement of this in membranes. Peptides offer higher tunability, making it easier to modify the peptide segment while keeping the oligourea segment the same. Furthermore, chimeric foldamers can adopt predicted secondary structures and have higher protease stability compared to natural peptides.

In this study, we designed, synthesized, and characterized three novel 15‐mer α‐peptide–oligourea chimeric foldamers. Two of them (HPU‐E, HPU‐N) independently form oligomeric fiber bundles in aqueous conditions. They exhibit high anion transport across lipid membranes. HPU‐N shows Cl^−^ selective transport via antiport mechanism, likely due to anion binding rather than size exclusion. X‐ray crystal structure of HPU‐N reveals assembly of helical bundles stabilized by Leu and Leu^U^‐like residues. Cryo‐electron microscopy (EM) shows self‐assembled channel structures incorporated into liposomes. Here, we demonstrated the use of α‐peptide–oligourea chimeras as Cl^−^ selective foldamers. We propose that similar self‐assembling and tunable chimeric foldamers are useful in designing various ion‐selective transporters. Our study shows that such synthetic novel scaffolds with desired functionality could have applications in biotechnology and biomaterials.

## 2 Results and Discussion

### 2.1 Design and Biophysical Characterization of Helical Peptide–Oligourea Chimeras

**Figure 1:**
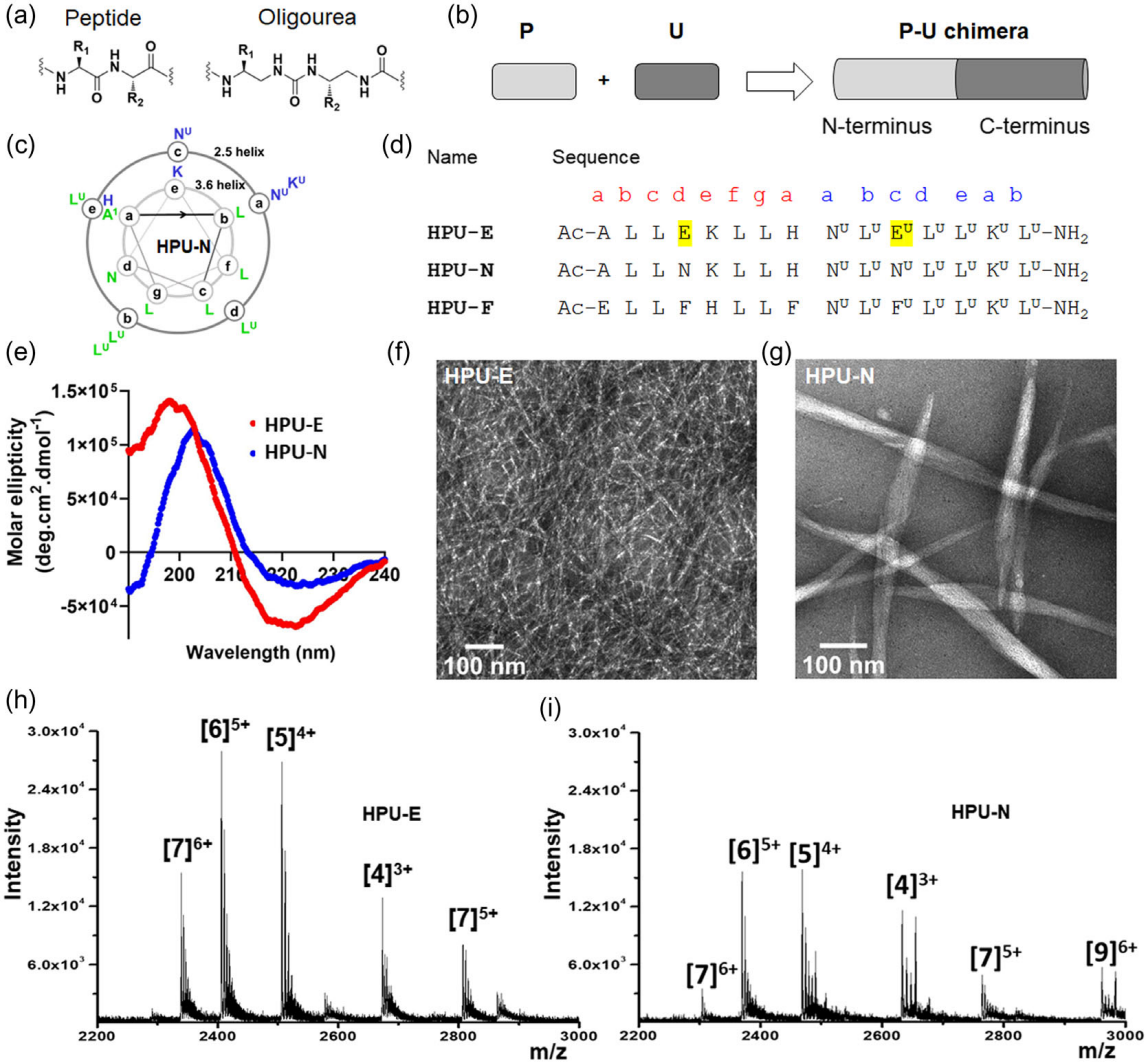
Design and characterization of α‐peptide–oligourea chimeras. a) Schematic representation of the α‐peptide and oligourea backbones. b) Representation of peptide (P) and oligourea (U) motifs in the chimeras. α‐Peptide and oligourea chains are colored gray and black, respectively. c) Helical‐wheel representation showing the side‐chain distribution pattern in HPU‐N as an example for the chimeras. d) Primary sequences of the three chimeras synthesized. HPU represents Helical Peptide–oligoUrea. Highlights indicate the residue difference among three sequences. Superscript ‘U’ represents urea residue. e) CD spectra of chimeras exhibit signature helicity in pure water. f,g) TEM shows self‐assembled fiber network of HPU‐E and fiber bundles of HPU‐N. h,i) electrospray ionisation mass spectrometry (ESI‐MS) analysis reveals heterogeneous oligomeric structures formed by both HPU‐E and HPU‐N. The CD, TEM, and ESI‐MS data for HPU‐F can be found in supplementary information.

Several artificial transmembrane ion channels have been designed using a coiled‐coil strategy in which individual helices wrap around each other to form a tubular helical bundle or channel‐like assembly.^[^ ^11^, ^27^, ^28^, ^29^, ^30^ ^]^ These higher‐order assemblies have shown accessible channel cavities with tunability.^[^ ^11^, ^28^, ^29^, ^30^ ^]^ Here, we designed and synthesized three (15 residues) amphiphilic α‐peptide–oligourea chimeras consisting of a hydrophobic and hydrophilic face, based on the ability of these hybrid oligomers to adopt well‐defined helical structures (**Figure** **1**a–d and S1, Supporting Information). From our initial design, they should self‐assemble into structures containing helical coiled‐coil dimers.^[^ ^23^ ^]^ The three chimeras were named HPU‐E, HPU‐N, and HPU‐F, where HPU stands for Helical Peptide–oligoUrea (Figure 1d). Several features were incorporated in the design of these chimeric ion channels. Our aim was to design amphiphilic helical sequences that could create a desired transmembrane self‐assembly with nonpolar hydrophobic sites that may facilitate its vertical insertion into a lipid membrane.^[^ ^30^, ^31^ ^]^ Accordingly, an array of eight isobutyl side chains (four Leu in the peptide part and four Leu^U^ in the oligourea part) was incorporated to have high hydrophobicity in the helix.^[^ ^30^ ^]^ The distribution and the number of polar residues (Asn, Asn^U^, His) were expected to provide control on the transmembrane aggregation and selective anion transport of the helices.^[^ ^30^ ^]^ The key differences among the three chimeric sequences are in the distribution of the hydrophobic and polar residues and in the nature of these residues. All chimeras were synthesized chemically by solid‐phase synthesis strategy and purified as reported previously (Scheme S1 and Figure S2–S4, Supporting Information).^[^ ^32^, ^33^ ^]^ At pH 7 HPU‐E is neutral, while HPU‐N is di‐cationic.

To gain insight into the folding propensity of the above‐mentioned chimeras, we first recorded their circular dichroism (CD) spectra in water at 100 μm. Because hybrid oligomers exhibit two types of chromophores (9 amides and 7 ureas in this study), their CD signature is expected to be more difficult to interpret than that of cognate α‐peptides and homo‐oligoureas. The canonical (*P*)‐2.5‐helix of oligoureas shows a characteristic CD signature with a maximum ≈200 nm and a relatively weak minimum ≈220 nm.^[^ ^23^, ^34^ ^]^ HPU‐E and HPU‐N exhibit helical conformation based on the CD data (Figure 1e and S5, Supporting Information) for the helical conformation of the peptide backbone (Figure 1e and S5, Supporting Information).^[^ ^35^ ^]^ They show maxima ≈200 nm and minima ≈220 nm, the characteristic bands of oligourea,^[^ ^23^, ^34^ ^]^ and canonical α‐peptide^[^ ^35^ ^]^ backbone. HPU‐E shows a deeper minimum at 222 nm than HPU‐N, which could indicate a more robust α‐helical backbone in HPU‐E. The maximum ≈200 nm is due to both peptide and oligourea backbones. HPU‐E and HPU‐N retained their helicity in 10 mm sodium acetate, pH 4.4 (Figure S5, Supporting Information). Self‐assembled nanostructures of chimeras were visualized by negative‐staining transmission electron microscopy (TEM) (Figure 1f,g and S6, Supporting Information). This study revealed that HPU‐E forms a network of fibers (diameter 5.6 ± 0.5 nm; Figure 1f), whereas HPU‐N forms extended fiber bundles, consisting of protofibrils, which have a diameter of 35.5 ± 5.5 nm in aqueous conditions (Figure 1f,g). The observed fiber bundles support the formation of higher‐order self‐assembled structures.^[^ ^22^, ^36^ ^]^

We further determined the oligomerization of chimeras in aqueous conditions by ESI mass spectrometry (Figure 1h,i, S7, and S8, Supporting Information), a conventional method to determine the stoichiometry of noncovalent assemblies.^[^ ^22^ ^]^ Mass spectrometry of HPU‐E and HPU‐N revealed the presence of heterogeneous multimeric species with predominant pentameric and hexameric species (Figure 1h,i, S7, and S8, Supporting Information). This observation further supports oligomerization by self‐assembly and formation of higher‐order structures mimicking biomolecules.^[^ ^22^ ^]^

We designed another chimera HPU‐F by incorporating phenylalanine (Phe) and phenylalanine‐type (Phe^U^) residues with the presumption that it may facilitate self‐assembly across lipid membranes (Figure 1d and S1, Supporting Information). HPU‐F is neutral at pH 7. A few additional variations were introduced in the sequence to improve the solubility (Ala1 → Glu1) and modification of the His position. CD analysis shows that a negative maximum at 222 nm is consistent with HPU‐F adopting a helical conformation (Figure S9a, Supporting Information). Interestingly, unlike the other two chimeras, this variant exhibited spherical nanostructures in TEM (Figure S9b, Supporting Information). The excess hydrophobicity and water insolubility of HPU‐F probably led to the formation of micellar structures in aqueous conditions. Mass spectrometry revealed lack of discrete oligomerization states (Figure S10, Supporting Information), indicating that HPU‐F does not form stable quaternary structures.

### 2.2 Solution Structures of the Chimeras by NMR

The folding of HPU‐E and HPU‐N in solution was determined using 1D and 2D NMR in pure H_2_O (Table S2 and S3, Figure S11–S15, Supporting Information). Urea and amide NHs are well separated with urea NHs showing more downfield chemical shifts than amide NHs in 1D NMR spectra. Further characterization of foldamers by 2D NMR revealed defined folding patterns and communication between peptide and oligourea segments. Nuclear overhauser effect spectroscopy (NOESY) spectra (Figure S11–S15, Supporting Information) revealed nonsequential *NN*(*i*, *i* + 1), medium‐range *αN*(*i*, *i* + 3), and *αN*(*i*, *i* + 4) nuclear Overhauser effects (NOEs) in the peptide segment, supporting a well‐defined *α*‐helical conformation. Similarly, *βN*(*i*, *i* + 2) and *βN*’(*i*, *i* + 2) NOE connectivities support the formation of a 2.5‐helical conformation in the oligourea segment. Furthermore *αN*(5,9), *αN*(7,10), and *αN*(8,11) NOE crosspeaks between the peptide (*α*CH) and oligourea segments (NH) are in good agreement with the propagation of the helical structure between the peptide and oligourea segments in the two chimeras (Figure S11–S15, Supporting Information).

### 2.3 Ion Transport Activity Across Lipid Bilayers

**Figure 2:**
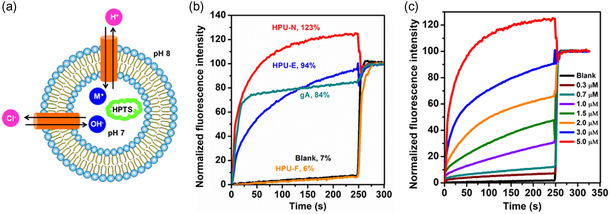
Ion transport activity of chimeras determined using HPTS assay. a) Schematic representation of LUV‐based HPTS assay. Phosphatidylcholine/phosphatidylserine (PC/PS ratio of 4:1) was used to constitute the LUV with internal/external buffer containing 100 mm NaCl. The internal pH was 7, and external pH was 8. HPTS (8‐hydroxypyrene‐1,3,6‐trisulfonic acid) shows increased fluorescence with increasing pH. b) Comparative ion transport activity of three chimeras at 5 μm. Lipid and foldamer concentrations were 100 and 5 μm, respectively. c) Concentration‐dependent increase of ion transport activity of HPU‐N across PC/PS (4:1) LUV.

The ion transport activity of the foldamers was assessed using a pH‐sensitive fluorescent dye 8‐hydroxypyrene‐1,3,6‐trisulfonate (HPTS) trapped in large phosphatidylcholine/phosphatidylserine (PC/PS, 4:1) unilamellar vesicles (LUV) having hydrodynamic radius of ≈58.5 ± 3.3 nm determined using dynamic light scattering (DLS) (**Figure** **2**a).^[^ ^37^, ^38^ ^]^ Both HPU‐N and HPU‐E at 5 mol% (lipid and foldamer concentrations were 100 and 5 μm, respectively) exhibit ion transport activity when a pH gradient is established across the membranes of the LUV, suggesting that they are inserted into the lipid bilayer.^[^ ^37^, ^38^, ^39^, ^40^, ^41^, ^42^ ^]^ Before disruption of HPU‐N LUVs by Triton X‐100, the emission intensity reached 23% higher than the maximum emission intensity at pH 8 (after disruption by Triton X‐100). This indicates that the intravesicular pH exceeded the extravesicular pH of 8.0 (Figure 2b).^[^ ^39^ ^]^ At 5 μm, HPU‐N showed ≈1.3 times higher ion transport activity than HPU‐E. We also observed that normalized fluorescence intensity of HPU‐N exceeds 100% before stabilizing due to higher pH inside the vesicle than external environment at this period. Substitution of two residues Glu and Glu^U^ with Asn and Asn^U^ facilitated higher ion transport by HPU‐N (Figure 2b). The third foldamer HPU‐F did not affect the emission intensity in this assay (Figure 2b), probably due to lack of distinct hydrophilic–hydrophobic regions^[^ ^30^ ^]^ and inability to form oligomeric ion channels. This suggests that an optimum balance between the sequence‐imposed hydrophobicity and hydrophilicity of HPU‐N might allow better incorporation as transmembrane channels across lipid bilayers.^[^ ^30^, ^41^, ^42^ ^]^

The ion transport activities of HPU‐E and HPU‐N were determined from 300 nm to 10 μm and 300 nm to 5 μm respectively. The data were normalized and plotted against monomer concentration and analyzed by Hill equation (Figure 2c, S16, and S17, Supporting Information).^[^ ^41^, ^42^ ^]^ HPU‐E showed EC_50_ (effective concentration of foldamer to obtain 50% activity^[^ ^41^, ^42^ ^]^) value of 3.91 ± 0.95 μm (Hill coefficient *n* = 1.7 ± 0.3), whereas HPU‐N showed lower EC50 value of 2.8 ± 0.1 μm (*n* = 2.5 ± 0.5). The Hill coefficient, *n* > 1, represents that the foldamer associates to form a dimer or supramolecular channel assembly (Figure S16 and S17, Supporting Information).^[^ ^38^ ^]^

### 2.4 OH^−^/X^−^ Antiport as Preferred Transport Mechanism

**Figure 3:**
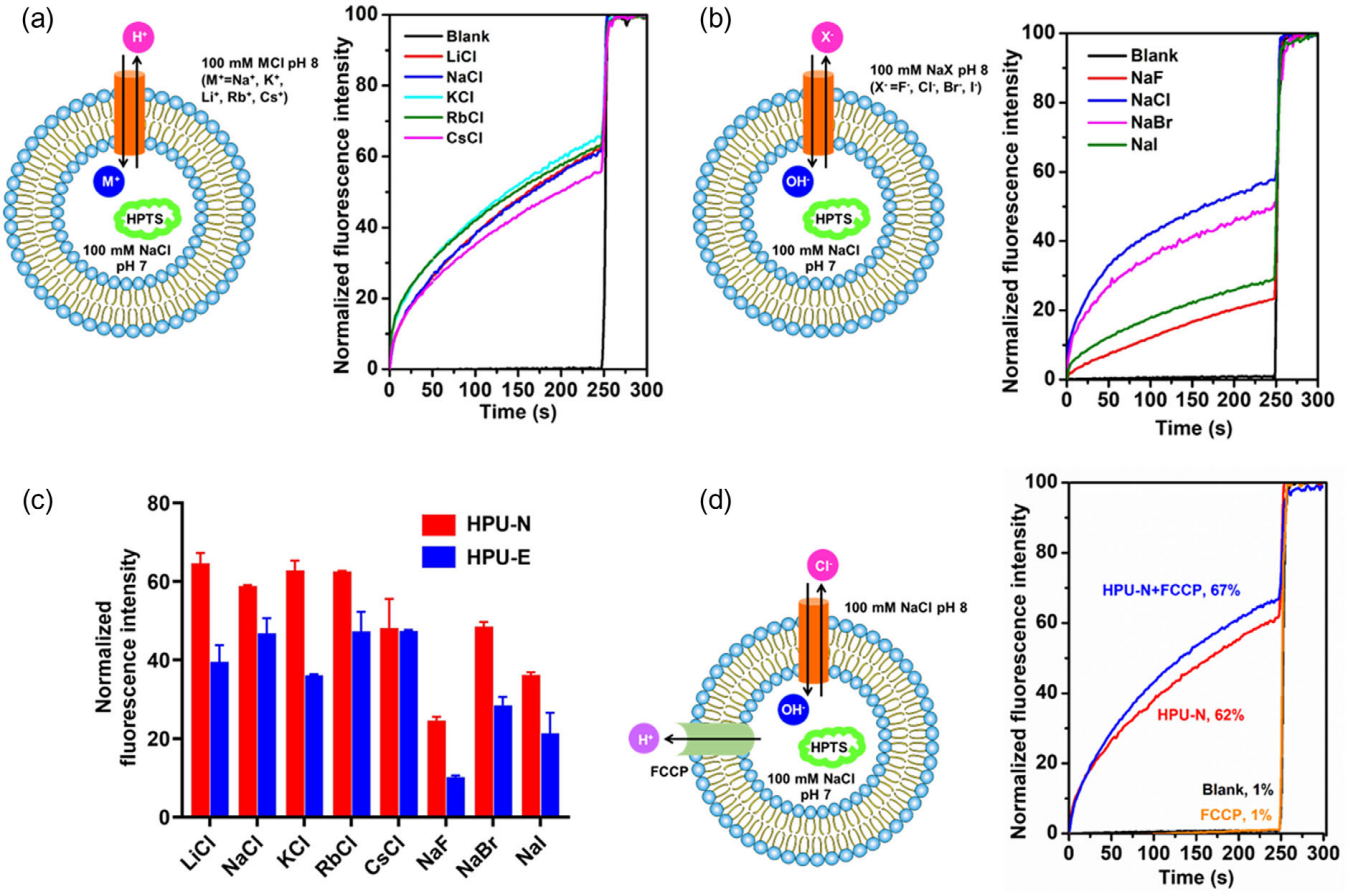
Cation/anion selectivity of HPU‐N. a) Schematic of HPTS assay for cation selectivity (left), and results of cation selectivity by HPTS assay (right) of HPU‐N (2 μm) after varying extravesicular cations MCl (where M^+^ = Li^+^, Na^+^, K^+^, Rb^+^, Cs^+^) with intravesicular NaCl. Lipid and HPU‐N concentrations were 100 and 2 μm, respectively, indicating 2 mol% foldamers. b) Schematic of HPTS assay for anion selectivity (left), and results of anion selectivity by HPTS assay (right) of HPU‐N (2 μm) after varying extravesicular anions NaX (where X^−^ = F, Cl^−^, Br^−^, I^−^) with intravesicular NaCl. c) The ion selectivity of HPU‐N (2 μm) and HPU‐E (2 μm) in the presence of different ions. d) Schematic of HPTS assay (left) in the presence of proton transporter FCCP and comparison of ion transport activity (right) of HPU‐N in the presence and absence of FCCP (2 μm). Lipid and HPU‐N concentrations were 100 and 2 μm, respectively, indicating 2 mol% foldamers.

The high ion transport activity of chimeras across the lipid bilayer encouraged us to investigate their ion selectivity. Varying the extravesicular cations and anions (100 mm MCl or NaX at pH 8) helps to understand the ion selectivity measured by the HPTS assay.^[^ ^37^, ^38^, ^41^, ^42^, ^43^ ^]^ The ion transport activities of HPU‐E and HPU‐N were determined in the presence of varying cations (Li^+^, Na^+^, K^+^, Rb^+^, Cs^+^) and anions (F^−^, Cl^−^, Br^−^, I^−^) (**Figure** **3** and S18, Supporting Information). Variation of extravesicular cations showed little difference in ion transport rate, suggesting minimal contribution from cations^[^ ^37^, ^38^, ^41^, ^42^, ^43^ ^]^ (Figure 3a,c and S18, Supporting Information). However, changing the extravesicular anions caused significant changes in the rate of ion transport,^[^ ^37^, ^38^, ^41^, ^42^, ^43^ ^]^ indicating that the ion transport is dependent on anions (Cl^−^ > Br^−^ > I^−^ > F^−^) (Figure 3b,c and S18, Supporting Information). The ratiometric changes in HPTS emission can occur by either cation exchange (M^+^/H^+^ antiport or M^+^/OH^−^ symport) or anion exchange^[^ ^37^, ^38^, ^41^, ^42^ ^]^ (X^−^/OH^−^ antiport or X^−^/H^+^ symport). Hence, we investigated the ion transport mechanism of chimeras in the presence of proton transporter carbonyl cyanide‐4‐(trifluoromethoxy)‐phenylhydrazone (FCCP) in PC/PS (4:1) liposomes by HPTS assay. FCCP allows H^+^ efflux from the vesicle to the extravesicular bulk environment. In the presence of FCCP, the ion transport activities of HPU‐E and HPU‐N were increased by 7% and 5%, respectively (Figure 3d and S19, Supporting Information). This indicates cooperativity of chimeras and FCCP indicates that X^−^/OH^−^ antiport is a preferred mechanism by which transport occurs (Figure 3d and S19, Supporting Information).^[^ ^37^, ^38^, ^43^ ^]^

### 2.5 Chloride‐Selective Transport by SPQ Assay and Anion Selectivity

**Figure 4:**
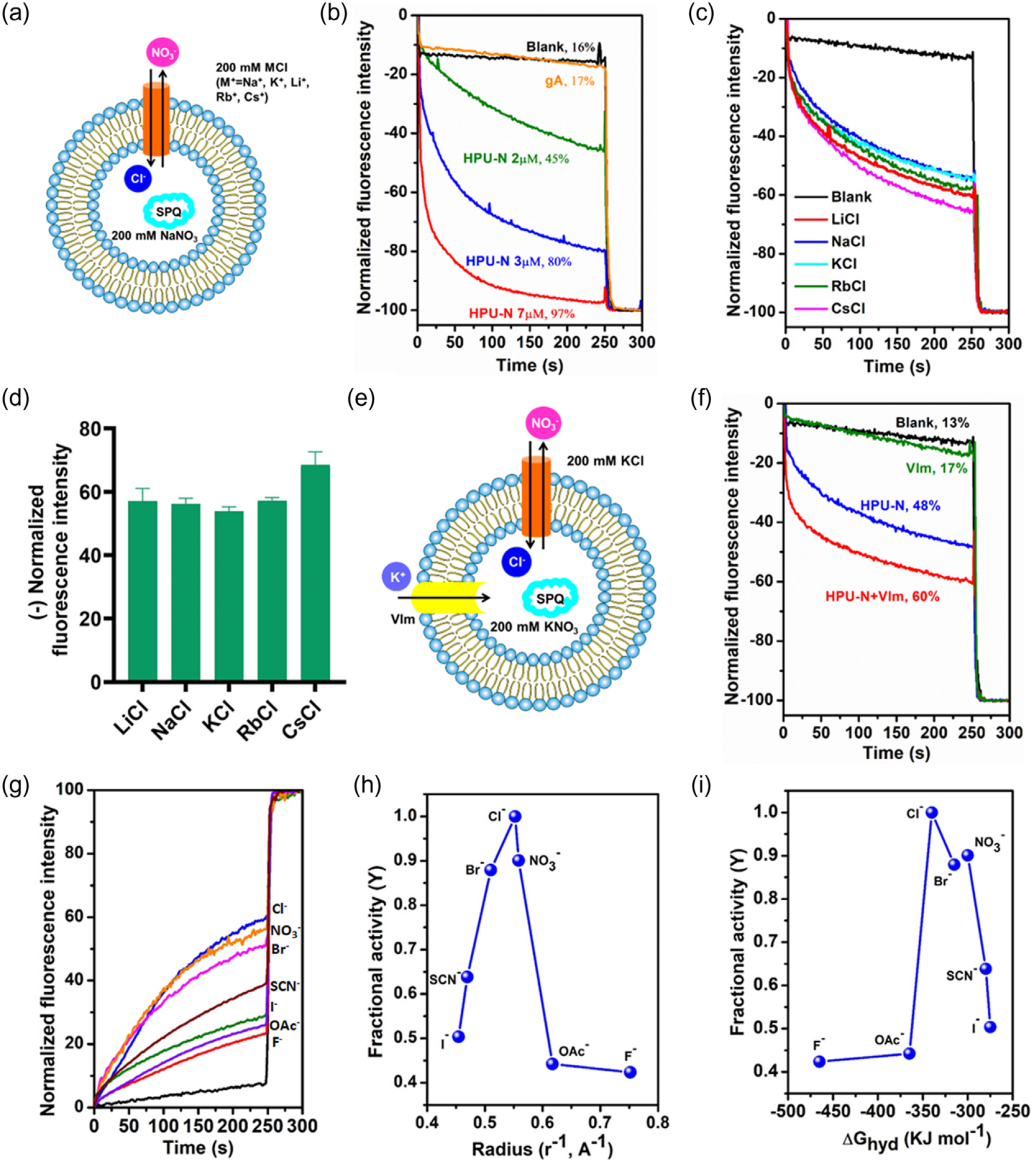
Chloride‐selective transport of HPU‐N by SPQ assay and anion selectivity of HPU‐N in the presence of different anions. a) Schematic of SPQ assay in the presence of intravesicular NaNO_3_ and extravesicular NaCl across PC/PS (4:1) liposome. b) Concentration‐dependent influx of Cl^−^ upon addition of HPU‐N. Influx of Cl^−^ leads to decrease in fluorescence intensity. Lipid concentration was 100 μm. c) Influx of Cl^−^ after varying extravesicular MCl ions (where M^+^ = Li^+^, Na^+^, K^+^, Rb^+^, Cs^+^) with intravesicular NaNO_3_ upon addition of 2.5 μm HPU‐N. Lipid and HPU‐N concentrations were 100 and 2.5 μm, respectively, indicating 2.5 mol% foldamers. d) Cation selectivity assay of HPU‐N shows that the decline in fluorescence intensity is independent of the cation species. e) Schematic of SPQ assay in the presence of valinomycin. f) The cotransport effect of valinomycin (Vlm, potassium transporter, 100 nm) on Cl^−^ transport activity of 2.5 μm HPU‐N. g) Anion selectivity trend of HPU‐N (2 μm) determined by HPTS assay with varying extravesicular X^−^ ions (where X^−^ = F^−^, Cl^−^, Br^−^, I^−^, NO_3_^−^, AcO^−^, SCN^−^) with intravesicular Cl^−^. Lipid and HPU‐N concentrations were 100 and 2 μM, respectively, indicating 2 mol% foldamers. Anion selectivity depicted as normalized emission intensity. h,i), Fractional activity Y (relative to Cl^−^) represented here as a function of reciprocal of anion radius (h) and hydration energy (i).

To support our previous observation, we further studied the ion transport activity through HPU‐N and HPU‐E using chloride‐selective dye 6‐methoxy‐N‐(3‐sulfopropyl) quinolinium (SPQ) across PC/PS (4:1) vesicles containing intravesicular NaNO_3_ and extravesicular NaCl (**Figure** **4**).^[^ ^38^, ^43^ ^]^ Concentration‐dependent quenching of fluorescence intensity was observed upon addition of HPU‐N, which indicates the influx of Cl^−^ (Figure 4b). The variation of extravesicular cations (M^+^ = Li^+^, Na^+^, K^+^, Rb^+^, Cs^+^) showed little difference in the rate of ion transport (Figure 4b). This not only indicates that cations are not transported at appreciable amounts, but also highlights the anion (Cl^−^) selectivity of the chimeras.^[^ ^38^ ^]^

This was further assessed using the potassium transporter valinomycin (Vlm) by SPQ assay. The Cl^−^‐selective transport of these chimeras in the presence of Vlm showed 12% increase in Cl^−^ influx by both HPU‐N and HPU‐E due to the strong cooperative effect of Vlm (Figure 4f and S20, Supporting Information). This observation not only suggests that Cl^−^ transport is faster than OH^−^ but also implies that X^−^/OH^−^ antiport is the preferred transport mechanism,^[^ ^37^, ^38^, ^43^ ^]^ which is in good agreement with our earlier observation with FCCP (Figure 3d and S19, Supporting Information).

We further investigated the anion selectivity by HPTS assay across PC/PS (4:1) vesicles (Figure 4g). The anion selectivity sequence was interpreted from the fractional activity Y (relative to Cl^−^) that was plotted against the reciprocal of anion radius and anion hydration energy (Figure 4h,i).^[^ ^38^, ^41^, ^42^, ^44^ ^]^ The relative rate of ion transport across membranes by HPTS assay may be directly comparable with the rate of ion flux from Goldman–Hodgkin–Katz analysis of planar bilayer conductance experiments.^[^ ^42^, ^44^ ^]^

The change of extravesicular anions revealed the trend of anion transport by HPU‐N as Cl^−^>NO_3_^−^ > Br^−^>SCN^−^ >I^−^ > AcO^−^>F^−^ (Figure 4g). This indicates a decrease in the rate of ion transport with increasing halide radius with the exception of F^−^.^[^ ^37^, ^42^, ^44^ ^]^ This could be explained by the favorable hydration energy of F^−^, unlike other halides in which the hydration energies vary linearly with the reciprocals of the bond lengths (Figure 4h,i).^[^ ^45^ ^]^ However, in the case of complex ions (AcO^−^, NO_3_^−^, SCN^−^), the transport rate does not follow the calculated ion radius, because it may depend on their distinct molecular shapes. This indicates that the effective anion selectivity of the channel depends on both anion hydration energy and ionic radius.^[^ ^29^, ^37^, ^41^, ^42^, ^44^ ^]^ The mannitol‐based Rosette ion channel exhibits analogous trends in anion selectivity, with Cl^−^ binding attributed to O–H^…^Cl^−^ interactions along the nanotube, in accordance with Eisenman's theory.^[^ ^37^ ^]^ HPU‐E showed a similar trend of anion selectivity (SCN^−^ > NO_3_^−^ ≥ Cl^−^>AcO^−^≥Br^−^>I^−^ > F^−^) (Figure S21, Supporting Information).^[^ ^37^, ^41^, ^42^, ^44^ ^]^ According to Eisenman's theory, ion selectivity trend is described as a balance between dehydration penalty and energy gain from ion binding to the transporter.^[^ ^42^ ^]^ This suggests that anion selectivity of chimeras is highly correlated with hydration energy and supports strong anion binding to the channel.^[^ ^29^, ^37^, ^41^, ^42^, ^44^ ^]^

### 2.6 Water Transport Through Chimera Channels

The high ion transport ability of chimeras tempted us to measure their water transport properties. The presence of polar residues in the chimeras could facilitate the H‐bonding interactions with water molecules and facilitate water transport through the channels.^[^ ^46^ ^]^ Therefore, we determined the water permeability of chimeras in PC/PS (4:1) lipid vesicles (pH 7.0) (Figure S22, Supporting Information). We used stopped‐flow technique^[^ ^47^, ^48^ ^]^ to measure the light scattering and analyze the fast kinetics of water flux. The light scattering traces indicate higher water transport ability of HPU‐N (*P*_f_ = 6.4 μm s^−1^) compared to HPU‐E (*P*_f_ = 2.5 μm s^−1^) (Figure S22, Supporting Information). However, the osmotic water permeability indicates a slow water transport through the channels compared to the reported values for foldamer channels and gramicidin (GA).^[^ ^23^ ^]^ Furthermore, the improved efficiency of HPU‐N is due to mutations of highly polar Glu^U^ with less polar Asn^U^ residues of HPU‐E. This was presumed to improve the higher water flow through the channels with fewer H‐bonding networks.

### 2.7 Ion Transport Assay Using Patch‐Clamp Technique

**Figure 5:**
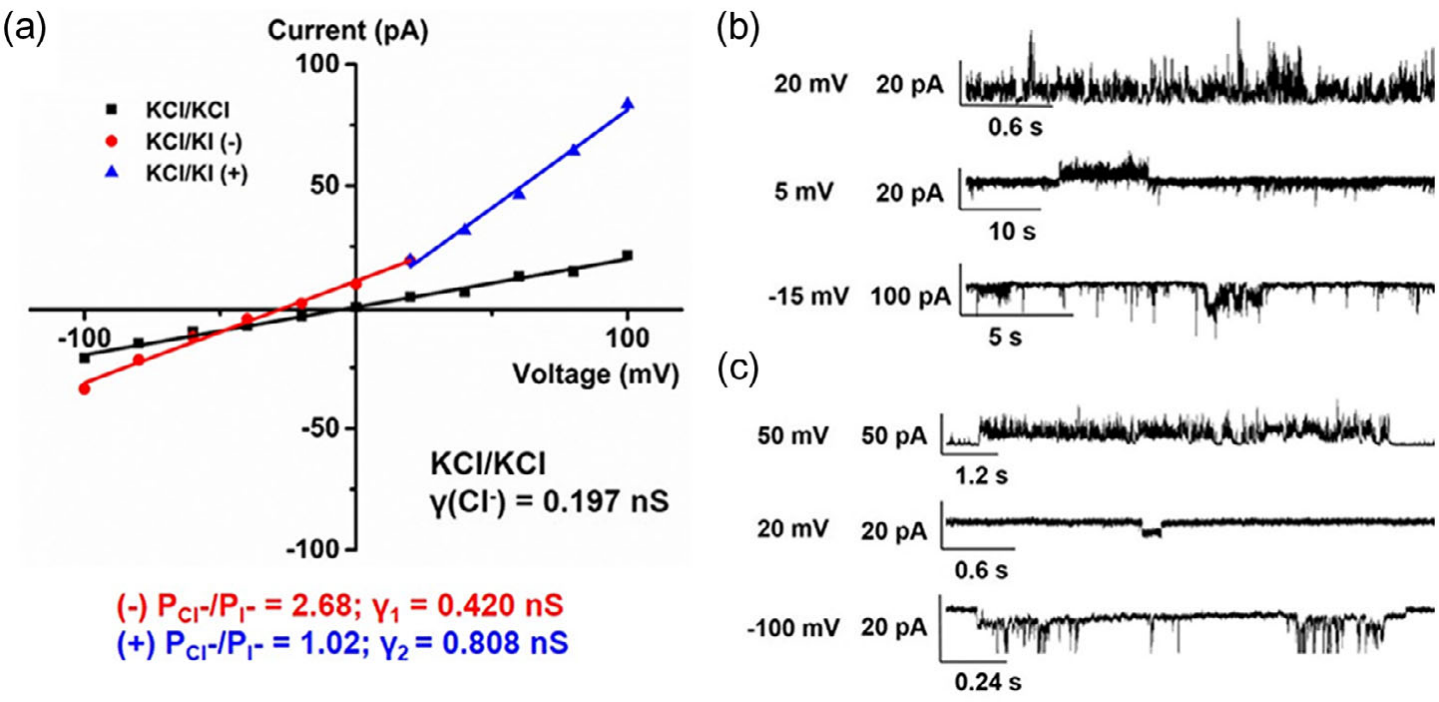
Ion transport assay of HPU‐N using patch‐clamp technique. a) *I–V* plots of HPU‐N and single‐channel current traces recorded. b) Symmetrical solution (*cis* chamber = 1 m KCl solution, trans chamber = 1 m KCl solution). c) Asymmetrical (*cis* chamber = 1 m KCl solution, trans chamber = 1 m KI solution) solution using PC/PS (4:1) lipids.

Ion transport through chimeric foldamer was investigated across the lipid bilayer using patch‐clamp techniques.^[^ ^46^, ^48^ ^]^ The calculated conductance value of 197 pS in KCl symmetrical solution suggested that HPU‐N displayed channel opening under the tested conditions (**Figure** **5**a,b and Scheme S2, Supporting Information). Subsequently, the ion transport selectivity of Cl^−^ over I^−^ was estimated in asymmetric solutions (Figure 5a,c). Channel opening was observed and a shift of −30.6 mV in the *I–V* plots was obtained, indicating that HPU‐N has a negative determined *ε*_rev_ value, and it transports Cl^−^ with a high selectivity ratio (PCl^−^/PI^−^ = 3.26) (Figure 5a). This is consistent with the fluorescence assay results, as shown in Figure 3 and 4.

### 2.8 Self‐Assembled Quaternary Structure of HPU‐N by X‐ray Crystallography

**Figure 6:**
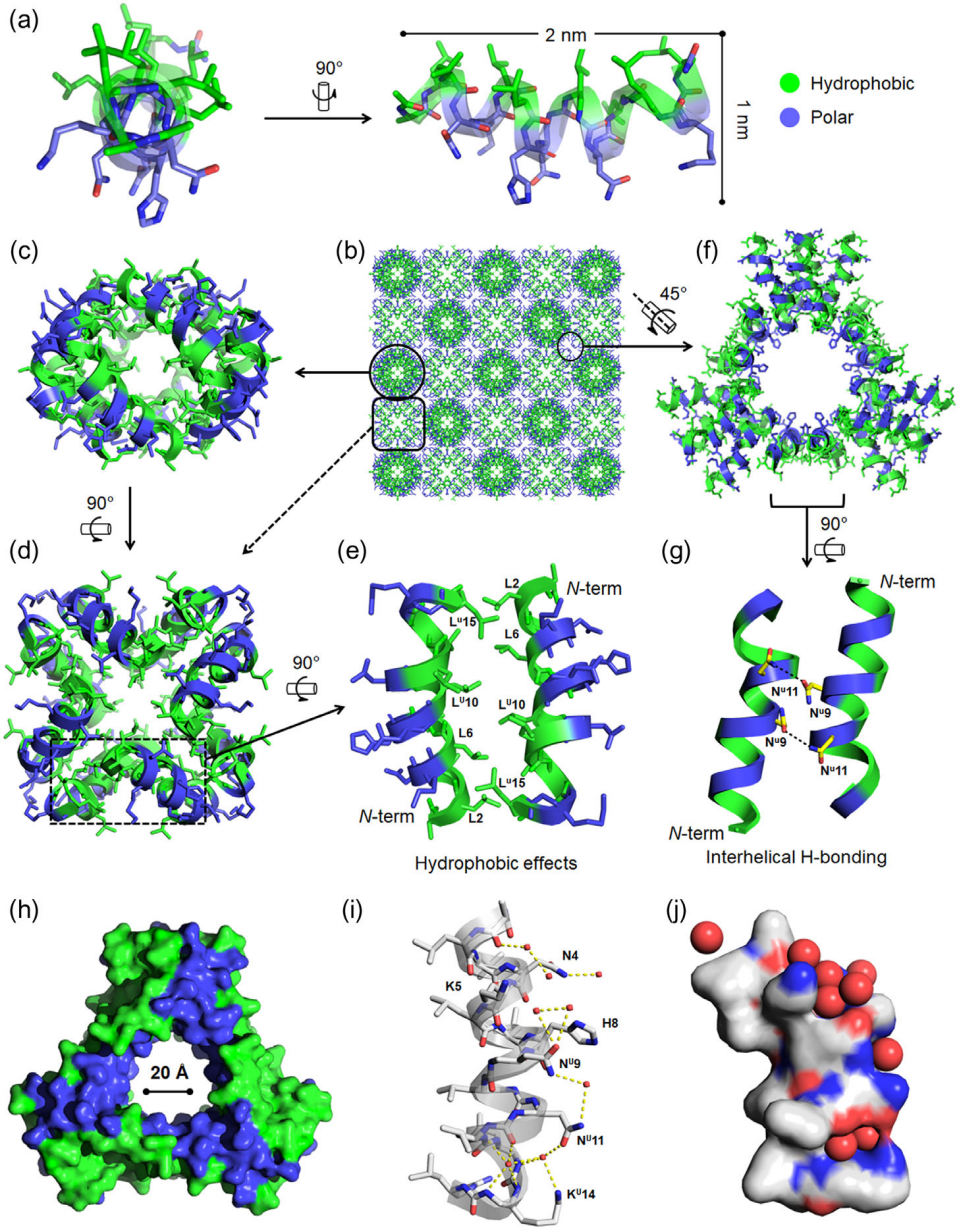
Quaternary structure of peptide–oligourea chimera HPU‐N. a) Crystal structure of HPU‐N shows that each monomer adopts a helical conformation throughout its chimeric backbone. It exhibits a bent helix geometry with distinct hydrophobic and polar surfaces. b) Crystal packing shows the presence of c,d) suprahelical, hydrophobic bundles and a f) hydrophilic pore. Figure S23, Supporting Information, includes additional detailed representation. (c,d) Octameric helical bundles are organized in two orientations in the crystal packing. Their hydrophobic residues form its core and hydrophilic residues are exposed to the surface. There are four pairs of dimers forming each octamer unit. e) Two chimeric helices are packed in antiparallel manner by hydrophobic interaction of Leu and Leu^U^ residues within each dimer. f) A hydrophilic pore is found between the octameric bundles. This pore is surrounded by three pairs of perpendicular helices and six pairs of parallel helices. This pore is lined by polar residues (Asn, Asn^U^, His, Lys, and Lys^U^). g) Each pair of these parallel helices is held together by interhelical H‐bonding between Asn^U^9‐Asn^U^11 as shown by dashed lines. h) The surface model of hydrophilic pore shows the distribution of polar and hydrophobic residues. Pore is built with mainly polar residues, and hydrophobic residues are present in the exterior surface. i) HPU‐N helix shows a bent geometry in the crystal packing, and insertion of water molecules may be responsible for this bent conformation. Red spheres represent water molecules. The polar residues, asparagine, and lysine form an intricate H‐bonding network with water molecules. j) The surface model shows that each HPU‐N helix forms pockets in the crystal packing and water molecules are occupied in this position. We anticipate similarly the anion selectivity of chimera regulated by the selection of ions within these pockets.

We obtained diffraction quality crystals of HPU‐N from 0.1 m MES pH 6.2, 0.6 m KCl at room temperature. We determined a 1.77 Å X‐ray crystal structure for HPU‐N (**Figure** **6**, S23–S27 and Table S4, Supporting Information). It confirms that HPU‐N adopts a helical conformation that spans the entire chimeric backbone with the oligourea segment forming the canonical 2.5‐helix and the peptide segment folded into an α‐helix. This chimeric helix contains a distinct and extended hydrophobic region composed of Leu and Leu^U^ residues and a hydrophilic face composed of Asn, Asn^U^, His, Lys^U^ resulting in overall amphipathicity (Figure 6a). In the crystal structure, HPU‐N shows self‐assembled, octameric hydrophobic pores (Figure 6b–d) interspersed with hydrophilic cavities (Figure 6b,f and S23–S27, Supporting Information). The wall surrounding the hydrophobic pores is made up of helical bundle pairs packed in an antiparallel fashion, held together by Leu–Leu^U^ hydrophobic contacts (Figure 6c–e and S24, Supporting Information). Four such dimeric units come together to create an octameric bundle (Figure 6c–e and S24, Supporting Information). Interspersed among the hydrophobic pores, we observed hydrophilic cavities (Figure 6f, S25 and S26, Supporting Information). They are formed by 6 parallel and 12 perpendicular helices (Figure 6f, S25 and S26, Supporting Information). The pore contains polar residues (Asn, Asn^U^, Lys, Lys^U^, His) (Figure 6h and S26, Supporting Information) and the pore is hydrated by a combination of water molecules that are both hydrogen bonded and free (Figure 6i,j and S27, Supporting Information). Interestingly, as previously observed in the case of an amphiphilic water‐soluble urea‐homo‐oligomer,^[^ ^49^ ^]^ the intramolecular H‐bond network of HPU‐N helix backbone is modified, but not broken. The insertion of several water molecules causes the helix to adopt a noncanonical bent geometry (Figure 6j). The solvent‐accessible Asn, Asn^U^ and Lys, Lys^U^ residues presumably play a role in anion binding (Figure 6j). This α‐helical structure of monomers provides useful insight into the self‐assembly properties of amphiphilic peptide–oligourea sequences and could serve to propose advanced models in membranes and first hints about the observed anion transport property of HPU‐N (see below). The foldamer pores are akin to the natural channel peptide alamethicin, forming a lipophilic exterior and hydrophilic interior with an inner pore diameter of 18 Å, comprising 8–9 monomers. It can transport cations and regulates action potentials in synthetic membranes.^[^ ^50^ ^]^ GA, another channel peptide, possesses a pore size of 4 Å, selectively facilitating the transport of monovalent cations across the membrane.^[^ ^51^ ^]^

### 2.9 Self‐Assembled Nanotube Structure of HPU‐N by Cryo‐Electron Microscopy

**Figure 7:**
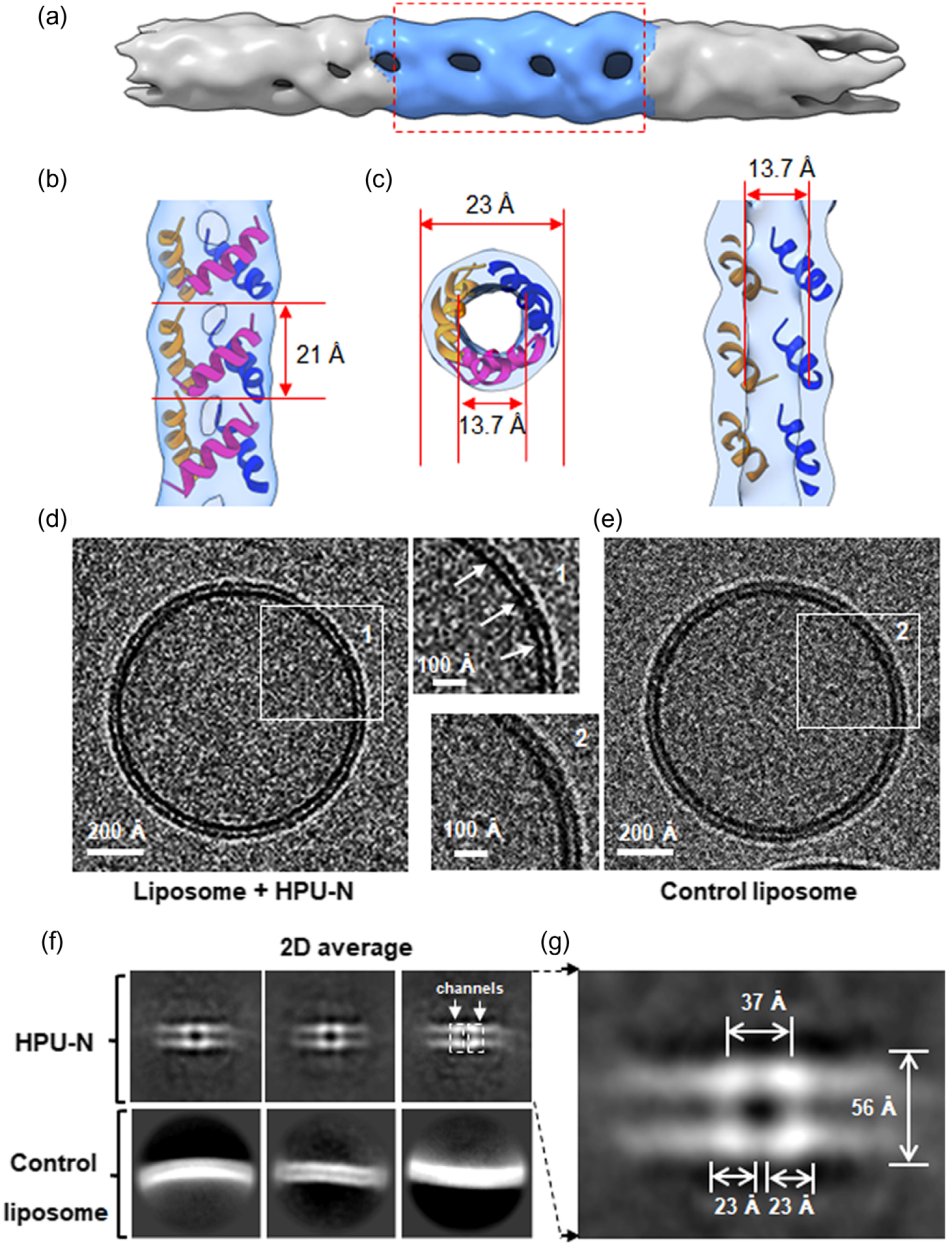
Cryo‐EM of the self‐assembled nanotube structure formed by HPU‐N. a) The final density map from 3D reconstitution of the nanotube formed by HPU‐N chimera. b) Surface view and model fitting of the boxed‐out part of cryo‐EM density map. Each turn (21 Å) could fit three helices of the foldamer. The surface map is shown with 60% transparency. c) In‐depth insights into the structural attributes were obtained through both longitudinal section and cross‐sectional perspectives, portraying the architecture of the HPU‐N channel. The contour level of the EM map shown is at 0.22 in chimera. d) Cryo‐EM micrograph of the HPU‐N embedded liposome (87.3 nm diameter), showing the lipid bilayer strata and the HPU‐N channels. Inset 1. A magnified region of liposome in (d) with channels identified by white arrows (see f for a clearer view). e) Cryo‐EM micrograph of the control liposome (88.5 nm diameter) without any HPU‐N channels, with a detailed view of the lipid bilayer structure. Inset 2. A magnified region of the control liposome in (e). f) Composite presentation of 2D averages delineating two adjacent HPU‐N channels (top right panel identifies two channels by dashed outlines. The two bright white spots in the upper and lower lipid layer correspond to one channel traversing the bilayer in these 2D average images), and the control liposomes (bottom) do not exhibit the distinct channel densities. Particles extracted through the application of the CryoSPARC algorithm were configured with a box dimension of 384 pixels, subject to sixfold binning. The distinctive channels characterized by discernible 2D class averages were systematically arranged in a descending order based on the particle count associated with each class. A selection of three out of eight 2D classes is depicted. g) Quantification of the dimensions of the two HPU‐N channels boxed in the top right panel of (f), along with the measurement of lipid bilayer strata (56 Å). Each foldamer channel is ≈23 Å in diameter, which matches the nanotube structure in panel (c). The distance between the centers of two adjacent channels is ≈37 Å.

To elucidate the assembly of the HPU‐N chimera and verify its channel formation in solution, we used single‐particle cryo‐EM. The EM map at 5.8 Å resolution shows C3 symmetry (**Figure** **7**a,b, and S28, Supporting Information). The foldamer assembles into a fibrous structure, measuring 23 Å in width and extending up to several micrometers in length. Each turn of this structure consists of three helical blocks, each ≈23 × 21 Å (Figure 7b,c). The EM density for each helical block suggests that it can accommodate a single‐chimeric foldamer helix. Given the limited resolution, we were unable to define the precise contact interface between the helices. Nevertheless, our cryo‐EM study of HPU‐N illustrates the formation of a channel with an inner diameter of 13.7 Å, which could facilitate ion transport.

Previously, we successfully used single‐particle cryo‐EM to study the insertion of another oligourea foldamer into liposomes and direct visualization of the water channels.^[^ ^23^ ^]^ Using the cryo‐EM images and data, we could define the dimensions of the channel assembly and calculate the number of channels per mm^2^. We used the same approach to investigate the channel formation of HPU‐N in a lipid environment; we introduced the foldamers into a liposome and subjected the sample to cryo‐EM studies (Figure 7d–g). We observed stretch‐like densities traversing the membrane and aligning uniformly across the foldamer‐reconstituted liposomes (Figure 7d), but not in control liposomes (Figure 7e). These observations suggest that HPU‐N is inserted into the lipid membrane forming uniformly distributed channels. Subsequent 2D classification demonstrated the insertion of foldamers as parallel channels traversing the membrane, while control liposomes displayed no such intramembrane structures (Figure 7f,g, and S29, Supporting Information). Consistent with the cryo‐EM images of the nanotube in solution (Figure 7a–c), each channel had a diameter of ≈23 Å, and two adjacent channels were separated by ≈14 Å with a pore‐to‐pore distance of 37 Å (Figure 7g). Considering the bilayer thickness of ≈60 Å, about three turns of the HPU‐N nanotube may traverse the membrane. The cryo‐EM analysis was carried out by embedding the foldamer in lipid bilayers, which yielded a greater abundance of native structures compared to those derived from solution‐phase crystallization conditions. Although the current cryo‐EM structure is of low resolution, it is evident that HPU‐N forms closely packed channels in the membrane, effectively facilitating ion transport. Based on the pore‐to‐pore distance between two neighboring HPU‐N channels, the estimated number of channels per μm^2^ at a 1:100 (foldamer to lipid) molar ratio will be 73 046. Also, the calculated net osmotic permeability (Pf) of 6.4 μm s^−1^ and the estimated single‐channel permeability was P1 = 1.06 × 10^−16^ cm^3^ s^−1^, and P2 = 3.5 × 10^6^ water molecules/s.

## 3 Conclusion

In summary, we have designed and synthesized three amphiphilic peptide–oligourea chimeras with two of them exhibiting anion transport activity across lipid membranes. Our design strategy to combine a short peptide chain and an oligourea segment in a single‐molecular strand resulted in a hybrid backbone that retains the ability to form a helical structure. CD, mass spectrometry, and TEM provided evidence for foldamer oligomerization and the formation of higher‐order structures. HPU‐E and HPU‐N form self‐assembled fiber bundles in aqueous conditions. Fluorescence‐based assays showed anion‐selective transport activity of HPU‐E and HPU‐N across lipid bilayers. The third foldamer synthesized, namely, HPU‐F, was inactive. These results highlight that increased hydrophobicity in HPU‐F affects the oligomerization and self‐assembly. Thus, a balance between hydrophobicity and hydrophilicity is necessary for self‐assembly and optimal insertion into the membrane. Moreover, HPU‐N exhibits significant Cl^−^ selectivity. Interestingly, the anion selectivity did not follow the common Hofmeister series or halide I topology. We speculate that this could be attributable to selective binding of anions within the foldamer channel independent of ion size. X‐ray crystallographic analysis of HPU‐N revealed a complex architecture and a hierarchical self‐assembly of amphiphilic chimeric α‐helical units. Cryo‐EM showed formation of similar‐sized nanotubes in both aqueous conditions and liposomes. The 23 Å nanotubes are formed by three chimeric foldamer helices per turn (21 Å height). These nanotubes have an internal pore of 13.7 Å and are properly inserted into liposomes allowing ion transport. Overall, this study shows that diverse and tunable α‐peptide–oligourea chimeras can be designed to form amphiphilic helices with the ability to self‐assemble and insert into membranes and could serve as lead molecules to develop ion channel‐based therapeutics in the future.

## 4 Experimental Section

4.1

4.1.1

##### Materials

All reagents for the synthesis of chimeras were procured from Sigma‐Aldrich unless stated otherwise. Lipids were obtained from Avanti‐polar lipids. Valinomycin, 8‐hydroxypyrene‐1,3,6‐trisulfonate (HPTS), 6‐methoxy‐ N‐(3‐sulfopropyl) quinolinium (SPQ), and carbonyl cyanide‐4‐(trifluoromethoxy)‐phenylhydrazone (FCCP) were obtained from Sigma‐Aldrich for fluorescence‐based assays. Carbon‐coated copper grid (300 mesh) for TEM was obtained from Electron Microscopy Sciences. Crystallization kits were obtained from Molecular Dimensions, Nextal, and Hampton Research. Milli‐Q water was used to prepare all buffers in biophysical experiments (CD, TEM, crystallization).

##### Synthesis of Foldamers

Chimeras were synthesized using standard solid‐phase peptide synthesis methods with activated succinimidyl (2‐azidoethyl)carbamate monomers.^[^ ^32^, ^33^ ^]^ NovaPEG Rink amide resin and a microwave reactor (CEM Discover) were employed for the synthesis. Detailed synthesis steps can be found in the supplementary information. All oligomers were purified and characterized by C18‐RP‐HPLC and mass spectrometry.

##### Circular Dichroism

CD experiments of chimeras were performed using J‐1100 CD spectrophotometer (Jasco). A quartz cuvette with path length of 1 mm was used. The spectra were measured in water and 10 mm sodium acetate, with pH 4.4 buffer of chimera concentration of 100 μm. Data were collected at 20 °C at 190–240 nm wavelength at 0.2 interval at a speed of 50 nm min^−1^ with three accumulations. After subtracting from the blank, data were plotted using OriginPro 8.5.

##### Transmission Electron Microscopy

Chimera samples (200 μm) were incubated at room temperature in 10 mm HEPES pH 7.0 for 3 days. A 5 μL sample was deposited on a glow discharge carbon‐coated copper grid (300 mesh) and incubated for 2 min. Excess sample was removed with filter paper, followed by negative staining using 1.5% uranyl acetate twice. The grid was then observed under a JEOL 1400 Flash TEM electron microscope.

##### NMR Spectroscopy

NMR spectra were recorded on a Bruker 800 MHz spectrometer equipped with a TXI cryo‐probe at 298 K. HPU‐E and HPU‐N were measured at 400 and 300 μm concentrations in 550 μL H_2_O containing 10% D_2_O. To assign proton chemical shifts, we used 2D ^1^H, ^1^H‐total correlation spectroscopy with a mixing time of 80 ms. For conformational identification we used 2D NOESY with a mixing time of 200 ms. Data were processed using nmrPipe and analyzed and plotted using NMRFAM‐Sparky 3.1.

##### Mass Spectrometry

Mass spectrometry was carried out using Synapt‐G2Si (Waters, UK). Both HPU‐E and HPU‐N were analyzed in 10 mm HEPES pH 7.0 at a concentration 200 μm with a pump flow rate of 300 μLmin^−1^ and source voltage 3 kVA. Furthermore, HPU‐N was analyzed in 20 mm ammonium acetate. The samples were eluted using methanol in the presence of 0.1% formic acid. The data analyses were performed by MassLynx and MaxEnt 1 software. Data were plotted using OriginPro 8.5.

##### Measurements of Ion Transport Activity: Preparation of PC/PS (4:1) Liposomes

Phosphatidylcholine (PC) and phosphatidylserine (PS) lipids were combined at a 4:1 molar ratio and mixed with methanol and chloroform (1:1) in a round‐bottom flask. The solvent was evaporated under reduced pressure using a rotavapor. After overnight drying at room temperature, the lipid film was rehydrated with a buffer (10 mm HEPES, 100 mm NaCl, pH 7.0) containing the pH‐sensitive dye 8‐hydroxypyrene‐1,3,6‐trisulfonic acid (HPTS) at 1 mm concentration. The mixture was vortexed for 30 min to release the lipids from the glass surface, followed by 9 freeze–thaw cycles (1 min in liquid nitrogen and 2 min heating at 55 °C in a dry bath). The resulting vesicle suspension was passed through a 0.2 μm polycarbonate membrane filter to obtain a uniform suspension of liposomes. Unencapsulated HPTS was removed by size‐exclusion chromatography using a Sephadex G‐50 column, yielding a 5 mm lipid stock. The size of PC/PS (4:1) liposomes was determined using DLS in Dynapro Wyatt technology. Before measurement, the liposomes were centrifuged at 12 000 rpm for 20 min. The reported measurements represented the average of three independent experiments.

##### Ion Transport Activity by HPTS Assay

HPTS‐containing liposomes at a concentration of 5 mm in a buffer containing 10 mM HEPES and 100 mM NaCl at pH 7.0 (40 μL) were added to a buffer solution (1.96 mL) with the same composition. A base pulse of 20 μL of 0.5 m NaOH was introduced to raise the pH from 7 to 8 before initiating the measurements. The measurements were carried out over 300 s, with continuous monitoring of emission at 510 nm using simultaneous excitations at 403 and 460 nm through a fluorescence spectrophotometer. At 50 s into the measurements, chimeras (5 μm) and gA (1 μm) were separately added to the system, and the curves were further observed. To obtain the maximum change in dye fluorescence emission, the vesicles were lysed using 10% Triton X‐100 after 300 s. Concentration‐dependent studies were performed over a range of 0.3–10 μm. The data were analyzed by plotting the ratiometric value of *I*_460_/*I*_403_ and normalizing it (IF) after the sample addition at 50 s. The normalization equation used was: IF = (*I*_t_–*I*_0_)/(*I*_∞_–*I*_0_) × 100, where *I*_t_ and *I*_0_ represent the ratiometric values of *I*_460_/*I*_403_ before the addition of Triton X‐100, and *I*_∞_ is the ratiometric value right after the addition of Triton X‐100. For data comparison and analysis, the time (X‐axis) was normalized between the point of foldamer addition (i.e., *t* = 50 s was normalized to *t* = 0 s) and the end point of the experiment (i.e., *t* = 300 s was normalized to *t* = 250 s). The EC50 and n values were obtained from the dose–response curve by fitting with the Hill Equation Y = 1/(1 + EC_50_/[C])^^*n*^, where Y represents the fractional activity (fractional emission intensity) at a given time, typically just before the addition of Triton X‐100. This fractional activity was used to compare the HPTS emission intensity under different conditions.

##### HPTS Assay for Cation and Anion Selectivity

The HPTS‐containing liposome suspension (40 μL, 5 mm in 10 mm HEPES, 100 mm NaCl pH 7.0) was added to the buffer (1.96 mL, 10 mM HEPES, 100 mm MCl, pH 7.0, M = Li^+^, Na^+^, K^+^, Rb^+^, Cs^+^) for cation‐selective assay and to the buffer (1.96 mL, 10 mm HEPES, 100 mm NaX, pH 7.0, X = F^−^, Cl^−^, Br^−^, I^−^, NO_3_^−^, SCN^−^, AcO^−^) for anion‐selective assay. A base pulse (20 μL, 0.5 m NaOH) was added to increase the pH from 7 to 8 before measurements. HPU‐N (2 μm) or HPU‐E (2 μm) was added at 50 s of measurements and the curves were monitored. The emission was monitored at 510 nm with excitations at both 403 and 460 nm simultaneously for 300 s using a fluorescence spectrophotometer. The liposomes were lysed at 300 s with 10% Triton X‐100 to obtain maximum change in dye fluorescence emission. The data was plotted as a ratiometric value of *I*_460_/*I*_403_ and normalized (*I*_F_) after addition of the sample at 50 s. *I*_F_ = *I*_t_–*I*_0_/(*I*_∞_–*I*_0_) × 100, where *I*_t_ and *I*_0_ are the ratiometric values of *I*_460_/*I*_403_ before addition of Triton X‐100 and *I*_∞_ is the ratiometric values right after addition of Triton X‐100. For data analysis and comparison, time (X‐axis) was normalized between the point of foldamer addition (i.e., *t* = 50 s was normalized to *t* = 0 s) and end point of experiment (i.e., *t* = 300 s was normalized to *t* = 250 s).

##### HPTS Assay in the Presence of FCCP

The HPTS‐containing liposome suspension (40 μL, 5 mm in 10 mm HEPES, 100 mm NaCl pH 7.0) was added to the buffer (1.96 mL, 10 mm HEPES, 100 mm NaCl, pH 7.0). A base pulse (20 μL, 0.5 m NaOH) was added to increase the pH from 7 to 8 before measurements. The FCCP (2 μm) and HPU‐N (2 μm) or HPU‐E (2 μm) were added at 50 s. The emission was monitored at 510 nm with excitations at both 403 and 460 nm simultaneously for 300 s using a fluorescence spectrophotometer. The liposomes were lysed with 10% Triton X‐100 to obtain maximum change in dye fluorescence emission at the end of the assay. The data was plotted as a ratiometric value of *I*_460_/*I*_403_ and normalized (*I*_F_) after addition of the sample at 50 s. *I*_F_ = *I*_t_–*I*_0_/(*I*_∞_–*I*_0_) × 100, where *I*_t_ and *I*_0_ are the ratiometric values of *I*_460_/*I*_403_ before addition of Triton X‐100 and *I*_∞_ is the ratiometric value right after addition of Triton X‐100. For data analysis and comparison, time (X‐axis) was normalized between the point of foldamer addition (i.e., *t* = 50 s was normalized to *t* = 0 s) and end point of experiment (i.e., *t* = 300 s was normalized to *t* = 250 s).

##### SPQ Assay for Chloride‐Selective Transport

PC/PS lipids were mixed at 4:1 molar ratio followed by the addition of methanol and chloroform (1:1) in round‐bottomed flasks and evaporated under reduced pressure using rotavapor. After drying overnight at room temperature, the film was rehydrated with 200 mm NaNO_3_ having SPQ (HPTS, 1 mm). The subsequent processing methods were as mentioned above (under preparation of PC/PS (4:1) liposomes), and this yielded 5 mm purified lipid stock.

##### SPQ Assay Using Different Cations

The SPQ‐containing liposome suspension (40 μL, 5 mm in 200 mm NaNO_3_) was added to the buffer (1.96 mL, 200 mm MCl, M = Li^+^, Na^+^, K^+^, Rb^+^, Cs^+^) and equilibrated for 2–3 min to stabilize the fluorescence intensity. HPU‐N (2.5 μm) and HPU‐E (2.5 μm) were added at 50 s and the curve was monitored after the addition. The emission was measured at 430 nm with excitations at 360 nm for 300 s using a fluorescence spectrophotometer. The liposomes were lysed with 10% Triton X‐100 to obtain maximum change in dye fluorescence emission at the end of the assay. The data was normalized (*I*_F_) after addition of the sample at 50 s and plotted. *I*_F_ = *I*_t_–*I*_0_/(*I*_0_–*I*_∞_) × 100, where *I*_0_ and *I*_t_ are initial fluorescence intensity and fluorescence intensity at time *t* and *I*_∞_ is the fluorescence intensity right after addition of Triton X‐100. For data analysis and comparison, time (X‐axis) was normalized between the point of foldamer addition (i.e., *t* = 50 s was normalized to *t* = 0 s) and end point of experiment (i.e., *t* = 300 s was normalized to *t* = 250 s).

##### SPQ Assay in the Presence of Potassium Transporter Valinomycin

The SPQ‐containing liposome suspension (40 μL, 5 mm in 200 mm NaNO_3_) was added to the buffer (1.96 mL, 200 mm KCl) and equilibrated for 2–3 min to stabilize the fluorescence intensity. At 50 s, valinomycin (0.1 μm) and HPU‐N (2.5 μm) or HPU‐E (2.5 μm) were added. The experiment and data processing were done as mentioned above.

##### Stopped‐Flow Assay for Water Permeability Measurements

Liposomes were prepared using a PC/PS mixture with a molar ratio of 4:1 dissolved in chloroform/methanol mixture (v/v = 1/1). The solvent was removed under vacuum and then rehydrated with HEPES buffer (10 mm, pH = 7.0) for 40 min. After hydration, the suspension was submitted to 5–10 freeze–thaw cycles (liquid nitrogen, water at 25 °C). Monodispersed LUV were obtained after extruding through 100 nm track‐etched filters (Whatman, UK) for 21 times and diluted with HEPES buffer solution to give 11 mm lipid stock solution (considering all the lipids have been incorporated). The average radius of the liposomes was determined using Zetasizer Nano (Malvern) system. 10 μL of 1 mm of the required chimera was injected into the corresponding hypertonic osmolyte (which contains 100 μL of 11 mm liposomes) to get a 5 μm final concentration in 2 mL total volume. Two independent experiments, each with at least three replicates, were carried out.

The light scattering experiments were performed on a stopped‐flow instrument (SFM3000 + MOS450. Bio‐Logic SAS, Claix, France) and data recorded at a wavelength of 345 nm, with 600 mm sucrose as the osmolyte (Δosm = 600 mOsmol/kg). Water permeability tests were conducted by quantifying the abrupt change of the liposome size measured as the variation in light scattering at 90°. Using the Rayleigh–Gans theory, data were calculated as the sum of two exponential functions (*y* = *at* + *b* + $\sum_{i=1}^{N=2}c_{i}e^{-k_{i}t}$) in Biokine software. The osmotic permeability (Pf) was calculated using the equation Pf = *k*/[(*S*/*V*_0_) × *Vw* × Δ*osm*], where k is the exponential coefficient of the change in light scattering and *S* and *V*_0_ are the initial surface area and volume of the liposomes, respectively; *V*w is the molar volume of water, and Δosm is the osmolarity difference.

##### Patch‐Clamp Technique for Measuring Ion Conductance and Ion Selectivity of Cl^−^ Over l^−^

PC/PS (4:1) lipids in CHCl_3_ were dried under N_2_ gas for 1 h and then redissolved in decane. Salt bridges (KCl/Agar) were placed in the chambers filled with KCl buffer (1 m, 10 mm HEPES, 10 mm Tris), attaching electrodes (Ag/AgCl), which were placed in KCl solution (1 M). Planar lipid bilayers were formed by brushing 0.2 μL of lipid‐containing n‐decane solution around the aperture, and a stable bilayer was obtained with a capacitance value ranging from 80 to 150 pF without applying any voltage. Single‐channel current traces were tested repeatedly at different voltages. The data were collected using ClampFit 10.3.1.5, and then analyzed in Origin, fitting to the linear equation of *y* = *a* + *bx* where slope b was the conductance value (*γ*), unit: nS.

For the measurement of ion selectivity ratio (PCl^−^/PI^−^), KCl buffer (1 m, 10 mm HEPES, 10 mm Tris) was used in the *cis* chamber and the trans chamber was still filled with KI buffer (1 m, 10 mm HEPES, 10 mm Tris). The data obtained were fit to the following Goldman–Hodgkin–Katz equation for calculation: *ε*_rev_ = *RT*/*F* × In(PCl^−^/Pl^−^), *R* = universal gas constant (8.314 J K^−1^ mol^−1^), *T* = 300 K, *F* = Faraday's constant (96 485 C mol^−1^), *P* = the permeability of sample for ions. ε_rev_ was determined by fitting to the linear equation of *y* = *a* + *bx*, where –intercept/slope (−*a*/*b*) was the reverse potential value (*ε*_rev_).

##### X‐ray Crystallography

The lyophilized power of chimera was dissolved in double‐distilled water to a final concentration of 10 mg mL^−1^. HPU‐N crystals were obtained at room temperature in 24‐well plates from 0.1 m MES pH 6.2, 0.6 m KCl. Crystals were soaked with crystallization solution supplemented with 25% glycerol as a cryo‐protectant before the data collection. Diffraction data were collected on beamline 23‐ID‐D of the Advanced Photon Source, Argonne National Laboratory. Diffraction data were integrated and scaled using X‐ray detector softwar^[^ ^52^ ^]^ and CCP4^[^ ^53^ ^]^ to a final resolution of 1.77 Å. Geometric restraints were generated using PRODRG^[^ ^54^ ^]^ with model building and restrained refinement performed in Coot^[^ ^55^ ^]^ and Refmac,^[^ ^56^ ^]^ respectively. Data collection and refinement statistics can be found in Table S4, Supporting Information. The structure was deposited in the CCDC with accession code 2222283.

##### Sample Preparation and Data Acquisition for Cryo‐Electron Microscopy of HPU‐N

The sample preparation process involved dispensing 2 μL of HPU‐N solution at a concentration of 1 mg mL^−1^ (in 10 mm HEPES buffer, pH 7.5) onto glow‐discharged Quantifoil holey copper grids (R 1.2/1.3, Cu 400 mesh) adorned with a monolayer of graphene oxide. The grids were blotted (6 s) under 100% relative humidity, followed by immediate plunge‐freezing in liquid ethane maintained at cryogenic temperatures through liquid nitrogen cooling in a FEI Vitrobot vitrification system (Gatan).

Cryo‐EM data acquisition was performed at the liquid nitrogen temperature, using a Titan Krios electron microscope (Thermo Fisher Scientific) equipped with a K3 Summit direct electron detector (Gatan) and a GIF Quantum energy filter. SerialEM4 (PMID: 16 182 563) was used for recording cryo‐EM movies in counting mode, using a slit width of 20 eV as set by the energy filter. These movies were captured at nominal magnification of 81 kx, which corresponded to a calibrated pixel dimension of 1.06 Å on the specimen plane. Each movie's total exposure duration amounted to 6 s, translating to a cumulative dose range of 45 electrons per [Å^2^], fragmented across 34 frames.

##### Cryo‐EM Data Processing of HPU‐N

The CryoSPARC software (PMID: 28 165 473) was used for data handling during EM image processing. Initial motion correction was carried out via MotionCor2 (PMID: 28 250 466) on dose‐fractionated movies gathered through the K3 Summit direct electron detector. For the determination of defocus parameters, the summed images derived from all frames of the movies, devoid of dose weighting, were subjected to CTFFIND4 (PMID: 26 278 980). Particle selection was executed using the blob‐picking approach with a box size of 256 pixels. A spectrum of methodologies, encompassing “2D classification”, “ab initio reconstruction”, “heterogeneous refinement”, “homogeneous refinement”, “nonuniform refinement”, and “helix refinement”, were enlisted for both 2D and 3D classification and refinement. Evaluation of overall resolutions was conducted employing the gold‐standard Fourier shell correlation at the threshold of 0.143. The assessment of local resolution was accomplished through “local resolution estimation”.

##### Single‐Particle Cryo‐EM to Determine Insertion of HPU‐N Channels into Liposomes

PC:PS (4:1) liposome samples, either featuring the presence of HPU‐N or controls without the chimeric foldamers, were applied onto glow‐discharged Quantifoil holey copper grids (R 1.2/1.3, Cu 400 mesh) before being promptly being immersed in liquid ethane maintained at cryogenic temperatures using liquid nitrogen within the FEI Vitrobot System. The cryo‐EM data were collected in the form of 34‐frame movie acquisitions, encompassing a magnification of 81 000×, facilitated by a Titan Krios electron microscope (Thermo Fisher Scientific). Subsequently, data processing was performed using the CryoSPARC software (PMID: 28 165 473). For both the HPU‐N and control liposome datasets, distinct subsets containing 41 230 particles and 45 524 particles, respectively, were isolated through the application of the “Blob picker” tool following the “Patch CTF estimation” procedure. The 2D averages for these subsets were obtained through the execution of the “2D classification” routine within the CryoSPARC platform.

## Conflict of Interest

The authors declare no conflict of interest.

## Author Contributions

G.G., R.M.K., P.P.K., and C.D. devised the project. C.D., G.G., S.H.Y., and M.P. designed and synthesized the molecular structures. C.D. and P.K. carried out the experimental work. C.D. obtained the crystals and diffraction data. C.D. and J.F. analyzed NMR data. J.L. and M.L. performed cryo‐electron microscopy studies. D.S. and M.B. carried out patch‐clamp work. C.D., G.G., D.S., M.B., R.M.K., and P.P.K. analyzed the experimental data. C.D. prepared manuscript draft. All authors participated in manuscript editing. Funding acquisition and supervision were done by G.G., M.B., R.M.K., and P.P.K.

## Supporting information

Supplementary Material

## Data Availability

The data that support the findings of this study are available in the supplementary material of this article.
