# Supplementary material for: Innovative Self‐Assembly of 15‐Mer Chimeric α‐Peptide–Oligourea Foldamers toward Cl−‐Selective Ion Channels
Source: Small Sci. 2024 May 30;4(8):2300352. doi: 10.1002/smsc.202300352 (PMC11935077; doi:10.1002/smsc.202300352)
Supplement: Supplementary file 1 — Supplementary Material [file SMSC-4-2300352-s001.pdf]

Supporting Information

Innovative self-assembly of 15-mer chimeric α-peptide-oligourea foldamers toward Cl^- selective ion channels

Chiranjit Dutta, Pannaga Krishnamurthy, Dandan Su, Jianwei Li, Sung Hyun Yoo, Gavin W. Collie, Morgane Pasco, Jingsong Fan, Min Luo, Mihail Barboiu,* Gilles Guichard,* R Manjunatha Kini,* Prakash Kumar.*

Table of Contents

Supplemental Experimental Procedures S4

1 Method S4

1.1 Solid phase synthesis of Chimera HPU-E, HPU-N, and HPU-F S4

1.1.1 Oligourea synthesis S4

1.1.2 Peptide Synthesis S4

1.2 X-ray crystallography S5

Scheme S1. Solid-phase synthesis of chimeric foldamers. S5

Table S1. Chimera sequences. S6

Figure S1. Helical-wheel representation of HPU-E and HPU-F S6

Figure S2: Solid phase synthesis of HPU-E. S7

Figure S3. Solid phase synthesis of HPU-N. S8

Figure S4. Solid phase synthesis of HPU-F. S9

Figure S5. CD analysis of HPU-E and HPU-N at 10 mM sodium acetate buffer pH 4.4. ..... S10

Figure S6. TEM images of HPU-E and HPU-N. S10

Figure S7. ESI-MS analysis of HPU-N in 20 mM ammonium acetate. S11

Figure S8. ESI-MS analysis of HPU-E in 20 mM HEPES buffer pH 7.0. ..... S12

Figure S9. Biophysical characterizations of HPU-F. S13

Figure S10. ESI-MS analysis of HPU-F. S13

Table S2. ^{1}H NMR assignments of HPU-E S14

Table S3. ^{1}H NMR assignments of HPU-N S14

Figure S11. Cross peak NOEs of HPU-N obtained from NOESY spectrum. S15

Figure S12. Partial TOCSY and NOESY spectra of HPU-N in H_2O at 298K. S16

Figure S13. Partial TOCSY and NOESY spectra of HPU-N in H2O at 298K. S17

Figure S14. Partial NOESY spectra of HPU-E measured in H_2O at 298K. S18

Figure S15. Partial NOESY spectra of HPU-E. S19

Figure S16. EC50 and Hill coefficient (n) of HPU-N was determined from the Hill analysis. S20

Figure S17. EC50 and Hill coefficient (n) of HPU-E was determined from the Hill analysis. S21

Figure S18. Ion selectivity of HPU-E. S22

Figure S19. Comparison of ion transport activity of HPU-E with carbonyl cyanide-4-(trifluoromethoxy)-phenylhydrazone (FCCP). S23

Figure S20. Comparison of ion transport activity of HPU-E with valinomycin (Vlm). S24

Figure S21. Anion selectivity is determined by fractional activity (Y). S25

Figure S22. Water permeability of HPU-N and HPU-E. S26
Calculation of number of channels from cryo-EM images S26
Single-channel water permeability calculation........ S26
Scheme S2. Schematic illustration of the patch-clamp technique for recording single channel currents. S27
Table S4. Data collection and refinement statistics for the X-ray crystal structure of HPU-N. S28
Figure S23. Crystal packing of HPU-N. S29
Figure S24. Crystal structure shows the helical bundle of HPU-N. S30
Figure S25. Crystal packing of HPU-N shows Hydrophobic interaction. S31
Figure S26. Pore residues in hydrophilic pore of HPU-N. S31
Figure S27. Crystal structure shows the water molecules in the Hydrophilic pore of HPU-E. S32
Figure S28. Single-particle cryo-electron microscopy investigation of the HPU-N chimera. S34
Figure S29. Flowchart of 2D average image processing of HPU-N in liposomes. S36

Supplemental Experimental Procedures

1 Method

1.1 Solid phase synthesis of Chimera HPU-E, HPU-N, and HPU-F

The chimeras **HPU-E, HPU-N**, and **HPU-F** were synthesized using a published protocol[1–3] in a polypropylene SPE tube (CEM) in a microwave reactor (CEM Discover) on NovaPEG Rink amide resin (loading rate: 0.45 mmol/g). Oligourea segments were synthesized first followed by elongation of the peptide part in automated peptide synthesizer.

1.1.1 Oligourea synthesis

The resin was swelled in dichloromethane (DCM) (3 mL) and dimethylformamide (DMF) (3 mL) for 2 h. Activated N_3-BB (150 µmol, 1.5 equiv.) and N,N-Diisopropylethylamine (DIPEA) (40 µL, 300 µmol, 3 equiv.) were dissolved in DMF (2 mL) and the solution was added to the reaction vessel. N_2 purge and bubbling in the vessel were performed. All microwave experiments were conducted at atmospheric pressure. The vessel was then placed inside the microwave reactor and irradiated (70 °C, 25 W, 20 min). After 20 minutes, the resin was filtered and washed with DMF (4x3 mL). This coupling step was performed twice. The resin was washed with a mixture of 1,4-dioxane:H_2O (i.e. reduction solvent, 7:3 v/v, 4x3 mL). Then, 1 M PMe_3 (trimethylphosphine) solution was added in THF (0.75 mL, 750 µmol, 10 equiv.) and reduction solvent (3 mL). The Staudinger reaction was performed to reduce the azide group under microwave irradiation (70 °C, 25 W, 15 min). After the reaction, the resin was filtered and washed with reduction solvent (3x3 mL) and DMF (3x3 mL). All steps were monitored by chloranil test.

1.1.2 Peptide Synthesis

The elongation of the peptide fragment was performed by classical automated SPPS following the last azide reduction of the oligourea chain on the resin. The coupling of N-Fmoc protected amino acids was performed in DMF using DIC (3 equiv.) and Oxyma (3 equiv.). The microwave was irradiated (25W, 75 °C, 10 min). This process was repeated for a second time. The resin was filtered and washed with DMF (2 mL). Fmoc deprotection steps were performed with 3 mL of 20% piperidine in DMF with microwave irradiation. After deprotection, the resin was filtered and washed with DMF (2x3 mL). After deprotection of the last α-residue of the sequence, N-terminal capping was carried using acetic anhydride (76 µL, 10 equiv.) and DIPEA (267 µL, 2000 µmol, 20 equiv.) in DMF under microwave irradiation (70 °C, 25 W, 10 min). After the reaction, the resin was filtered and washed with DMF (4x3 mL).

Before the cleavage, the resin was moved to the syringe with a filter and washed with DMF (4x3 mL) and DCM (5x2 mL). The cleavage from the resin was performed by treatment with trifluoracetic acid (TFA) (95%) /TIS (2.5%)/H_2O (2.5%) (total volume 4 ml) for 4 h. Resulting filtrates were evaporated. Peptide-oligourea chimera was precipitated in diethyl ether, then dried. The crude solid was dissolved in acetonitrile/deionized water solution (CH_3CN:DW=1:9) and lyophilized. Analytical RP-HPLC checked purity (C18 column,

method: 10-100% B/10 min, A: Milli-Q H_2O + 0.1% TFA, B: ACN + 0.1% TFA, flow=1mL/min, T=50 °C, λ=214 nm). Prep C18-RP-HPLC purified the product (Macherey-Nagel Nucleodur 100-5 C18ec column, method: 40-100% B/20 min, A: Milli-Q H_2O + 0.1% TFA, B: ACN + 0.1% TFA, flow 20 mL/min, λ=214 nm). The counterion was exchanged by lyophilizing powder with HCl solution three times (1. 0.05N $HCl_{(aq)}$, 2. 0.1N $HCl_{(aq)}$, 3. MQ H_2O).

1.2 X-ray crystallography

The lyophilized powder of chimera was dissolved in double distilled water to a final concentration of 10 mg/mL. Crystallization trials were performed using sparse screening kits (Cation suite from Nextal, Proplex, MemChannel, MemTrans, Morpheus, Morpheus II, Structure screen 1 & 2 from Molecular Dimensions, Crystal screen 2, PEG/Ion from Hampton research) in standard sitting drop in 96-well plate at room temperature. The crystals observed in these conditions were further grown in 24-well plates by standard hanging drops method for the optimization. A drop generally consists of 0.5 µL foldamer solution and 0.5 µL crystallization regent and incubated at room temperature as well as at 16 C° incubator. **HPU-N** crystals obtained at room temperature in 24-well plates from several conditions such as 1) 0.1M MES pH 6.2, 0.6 M KCl, 2) 0.1M Tris pH 8.5, 0.15 M $CaCl_2$, 3) 0.1M NaAcet pH 4.8, 0.3 M $MgCl_2$. The diffraction quality HPU-N crystals obtained from a crystallization reagent composed of 0.1M MES pH 6.2, 0.6 M KCl in 1 month at room temperature.

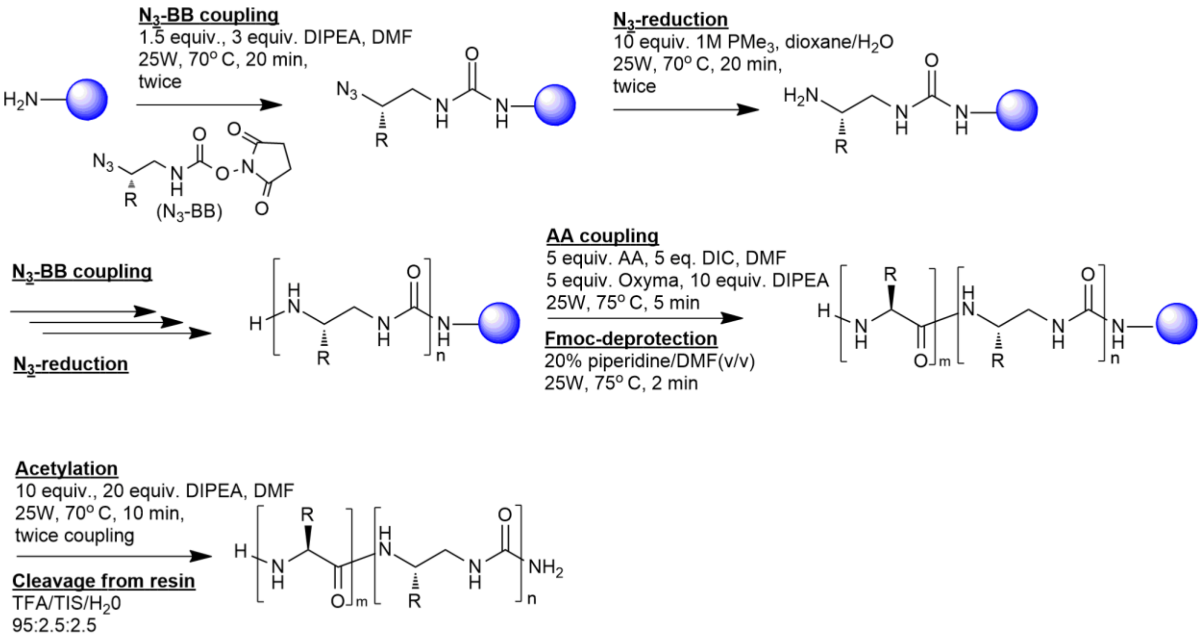

Scheme S1. Solid-phase synthesis of chimeric foldamers.

Table S1. Chimera sequences.

Foldamer Name	Sequence	Expected M.W. (Da)	Observed M. W. (Da)
**HPU-E**	Ac-A L L E K L L H N^U L^U E^U L^U L^U K^U L^U –NH_2	2004.51 Da	2005.41 Da ($[M+H]^+$), 1003.23 Da ($[M+2H]^{2+}$), 669.16 Da ($[M+3H]^{3+}$), 502.13 Da ($[M+4H]^{4+}$)
**HPU-N**	Ac-A L L N K L L H N^U L^U N^U L^U L^U K^U L^U –NH_2	1974.49 Da	1975.43 Da ($[M+H]^+$), 988.24 Da ($[M+2H]^{2+}$), 659.17 Da ($[M+3H]^{3+}$), 494.63 Da ($[M+4H]^{4+}$)
**HPU-F**	Ac-E L L F H L L F N^U L^U F^U L^U L^U K^U L^U –NH_2	2117.67 Da	1059.67 Da ($[M+2H]^{2+}$), 706.83 Da ($[M+3H]^{3+}$, 530.50 Da ($[M+3H]^{3+}$)

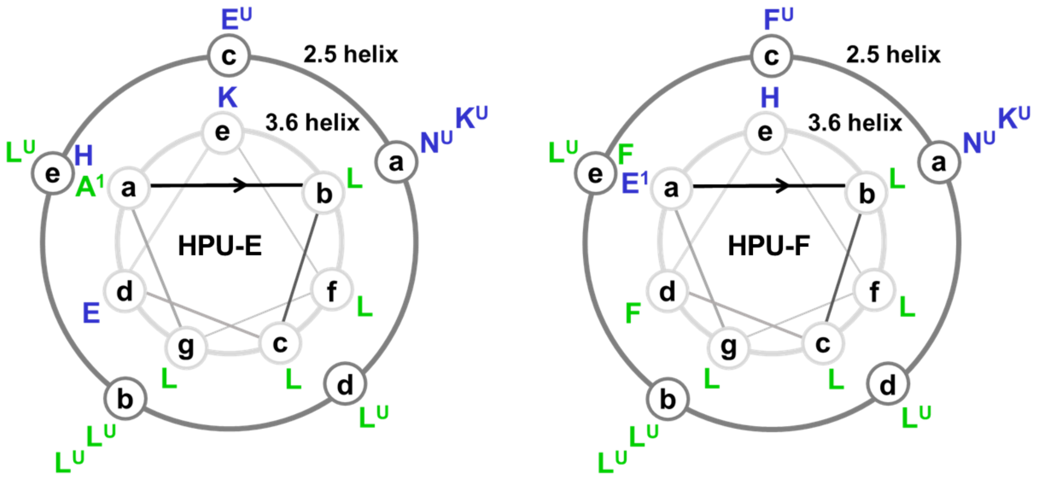

Figure S1. Helical-wheel representation of HPU-E and HPU-F showing the side chain distribution pattern.

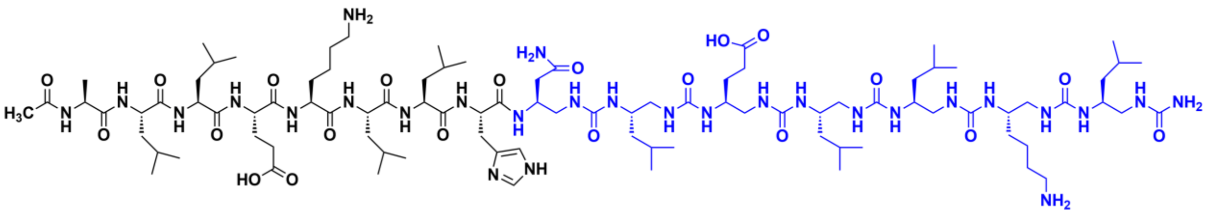

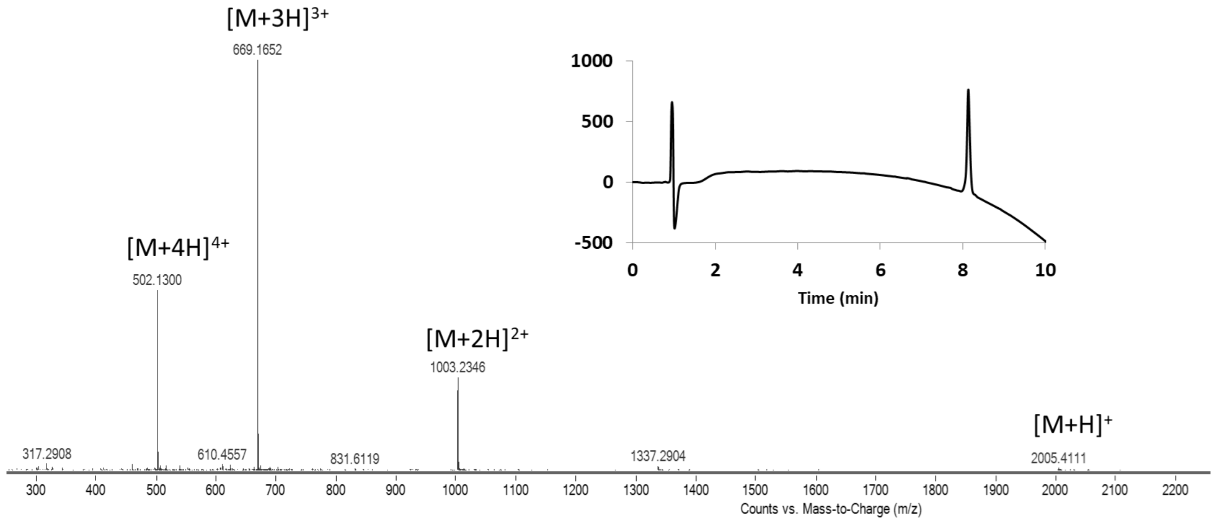

Figure S2: Solid phase synthesis of HPU-E. Chemical structure and ESI-MS spectrum of **HPU-E** foldamer. Expected M.W. 2004.51 Da; Obs. M.W. 2005.41 Da ($[M+H]^+$), 1003.23 Da ($[M+2H]^{2+}$), 669.16 Da ($[M+3H]^{3+}$), 502.13 Da ($[M+4H]^{4+}$). (In set) analytical HPLC profile of purified **HPU-E** foldamer is shown (t_R=8.14 min).

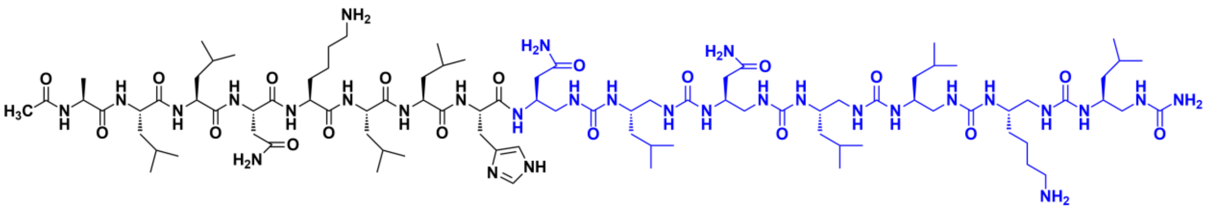

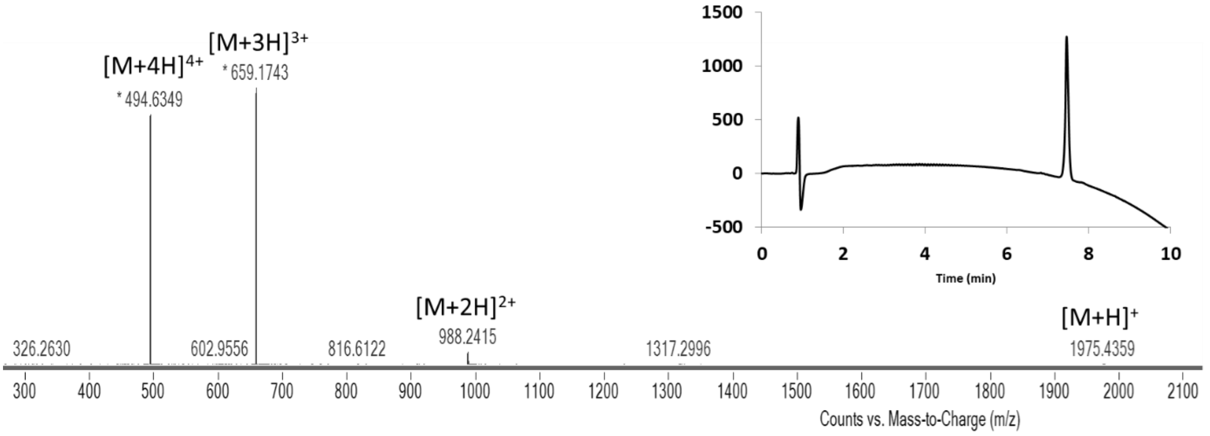

Figure S3. Solid phase synthesis of HPU-N. Chemical structure and ESI-MS spectrum of **HPU-N** foldamer. Expected M.W. 1974.49 Da; Obs. M.W. 1975.43 Da ($[M+H]^+$), 988.24 Da ($[M+2H]^{2+}$), 659.17 Da ($[M+3H]^{3+}$), 494.63 Da ($[M+4H]^{4+}$). (In set) The analytical HPLC profile of purified **HPU-N** foldamer is shown (t_R=7.0 min).

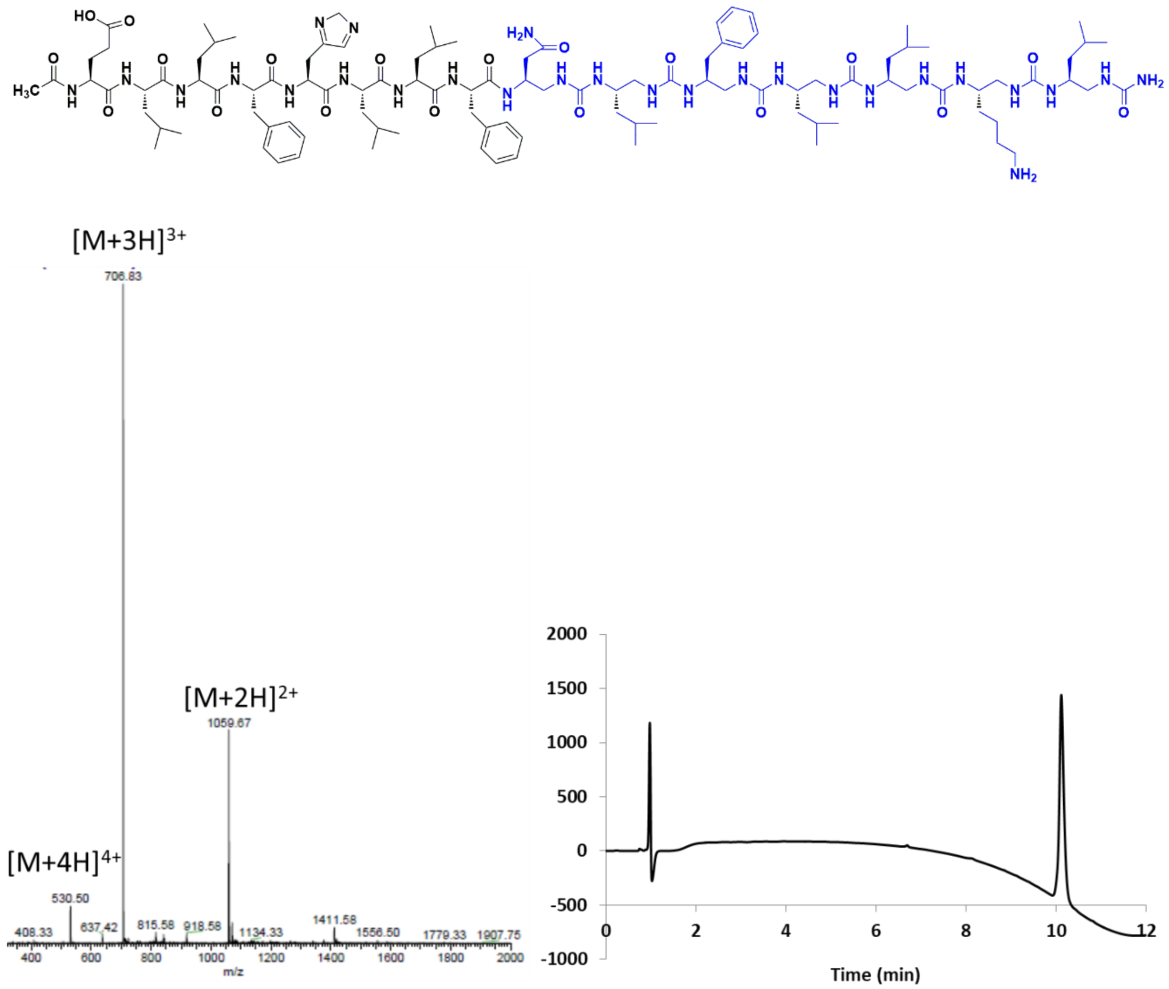

Figure S4. Solid phase synthesis of HPU-F. Chemical structure and (left) ESI-MS mass spectrum of **HPU-F** foldamer. Expected M.W. 2117.67 Da; Obs. M.W. 1059.67 Da ($[M+2H]^{2+}$), 706.83 Da ($[M+3H]^{3+}$, 530.50 Da ($[M+3H]^{3+}$). (Right) analytical HPLC profile of purified **HPU-F** foldamer is shown (t_R=10.13 min).

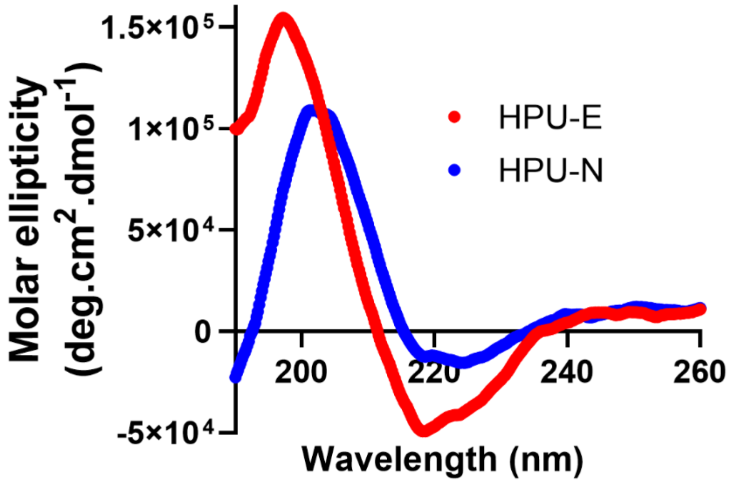

Figure S5. CD analysis of HPU-E and HPU-N at 10 mM sodium acetate buffer pH 4.4. The negative maxima peak around 222 nm represents the characteristic peaks for helical peptide-oligourea chimera.

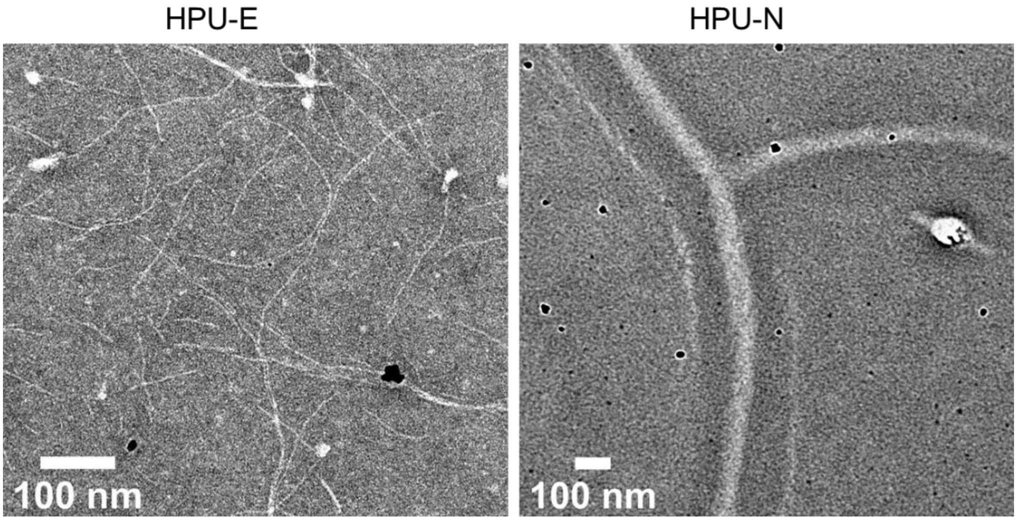

Figure S6. TEM images of HPU-E and HPU-N. At 10 mM sodium acetate, pH 4.4, both show fiber assemblies. However, **HPU-E** showed a fiber network whereas **HPU-N** exhibited large fiber bundles.

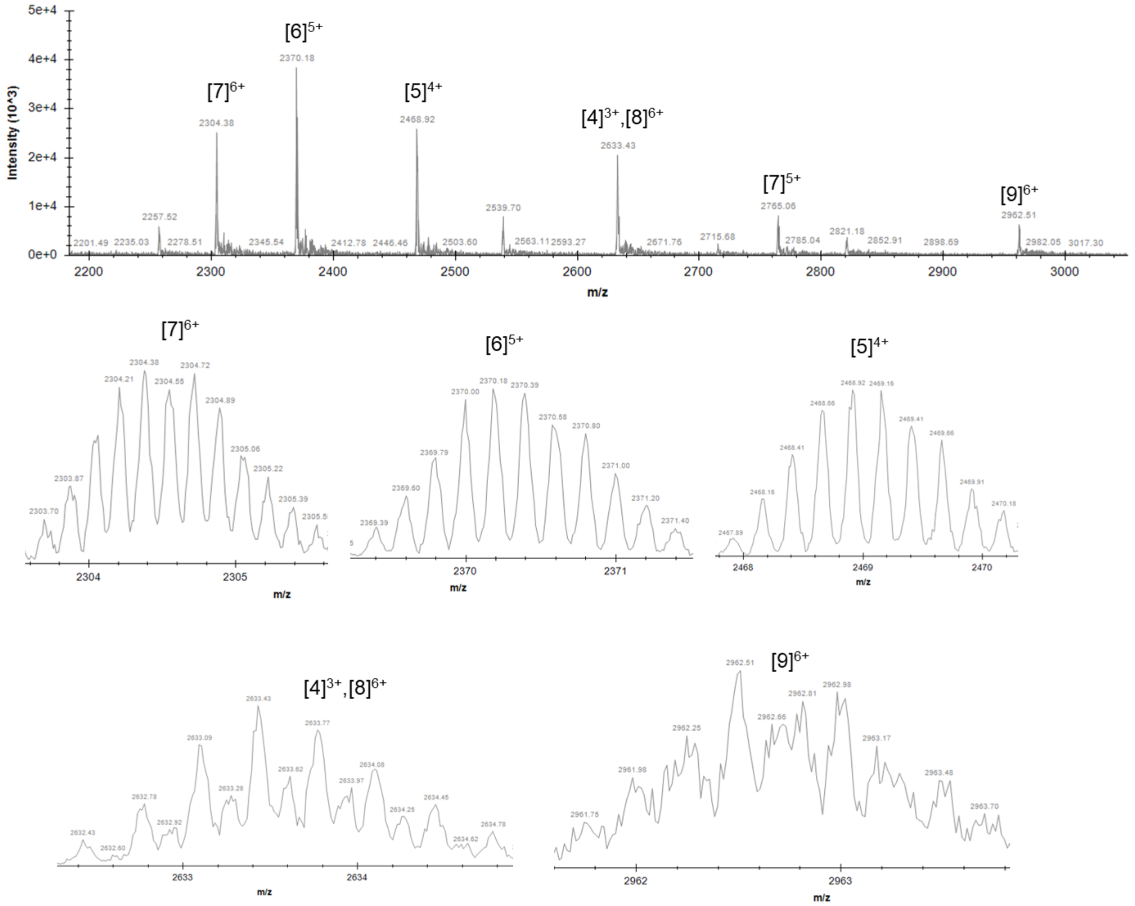

Figure S7. ESI-MS analysis of HPU-N in 20 mM ammonium acetate. The spectrum shows characteristic peaks for oligomerization with a stoichiometry of heterogeneous oligomers. The concentration of **HPU-N** was 200 µM.

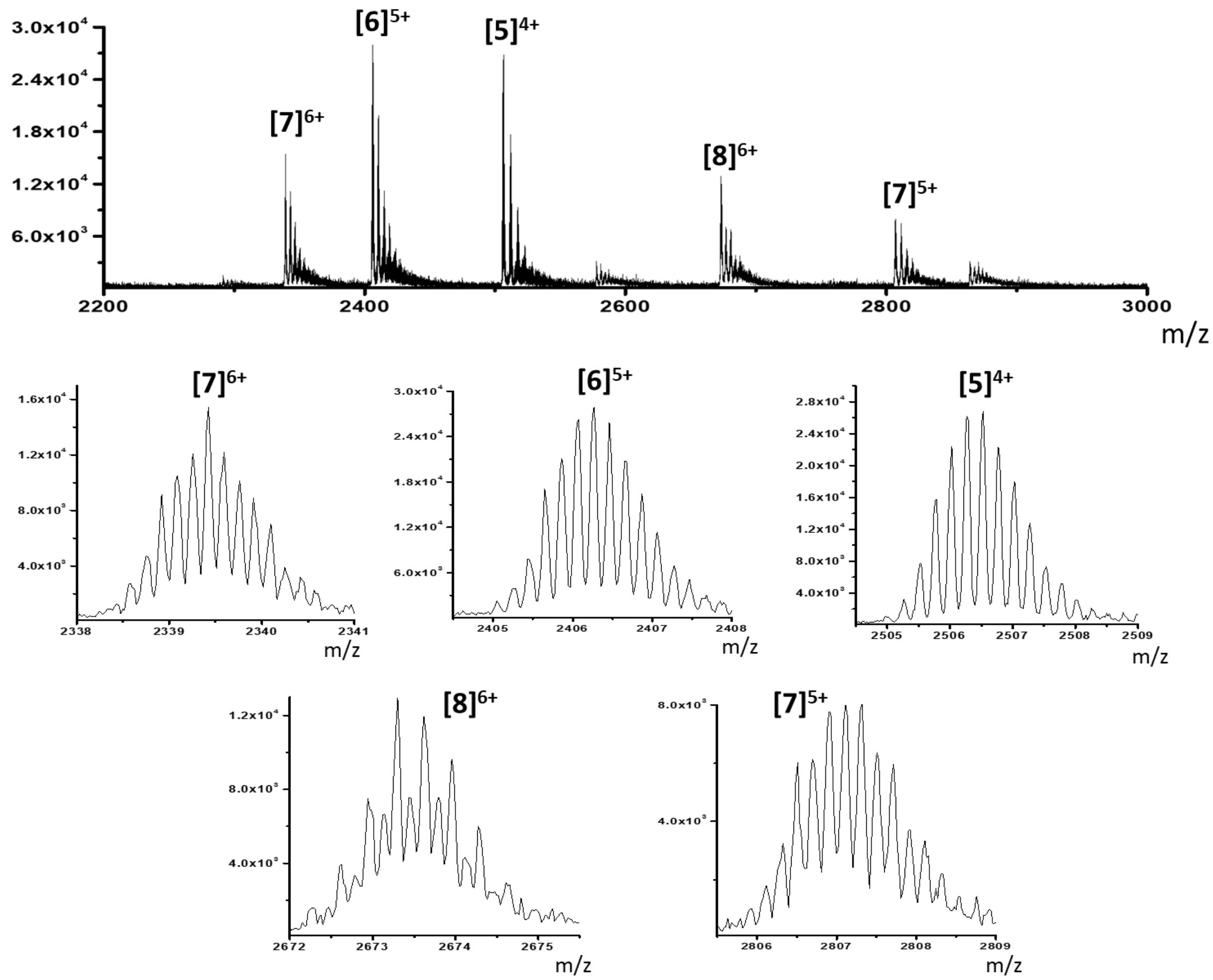

Figure S8. ESI-MS analysis of HPU-E in 20 mM HEPES buffer pH 7.0. The spectrum shows characteristic peaks for oligomerization with a stoichiometry of heterogeneous oligomers. The concentration of **HPU-E** was 200 µM.

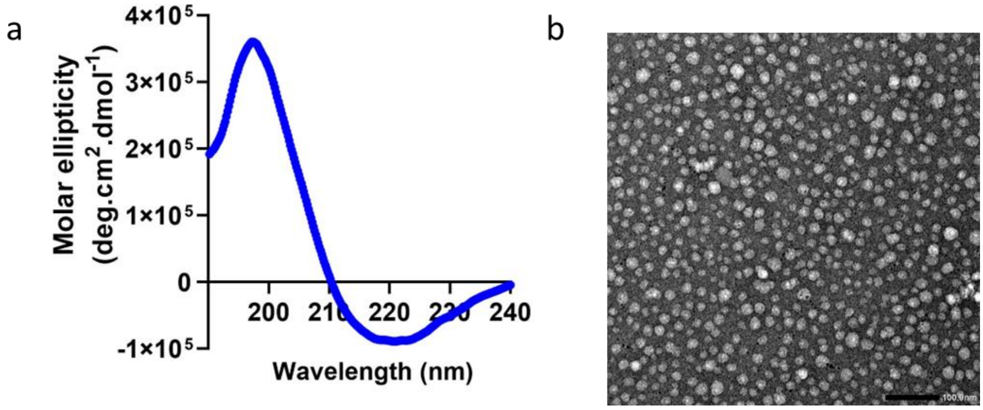

Figure S9. Biophysical characterizations of HPU-F. a) CD analysis of **HPU-F** in methanol and water mixture (1:1). b) **HPU-F** revealed spherical nanostructures. The scale bar represents 100 nm.

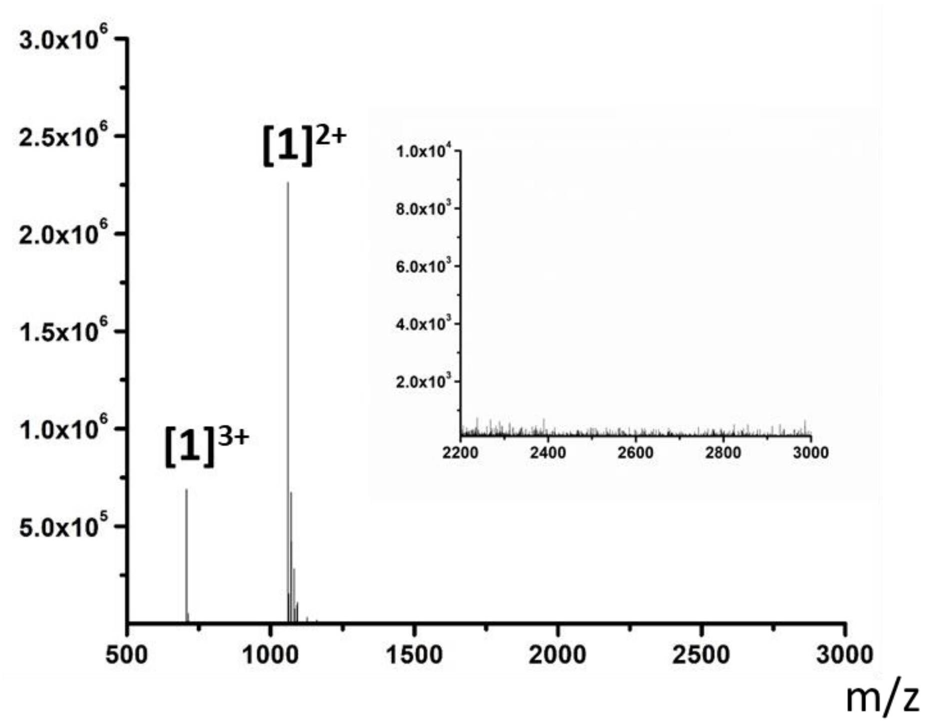

Figure S10. ESI-MS analysis of HPU-F. No characteristic oligomeric peak was observed in mass spectrometry, suggesting that it remained a monomeric species in solution.

Table S2. ^{1}H NMR assignments of HPU-E

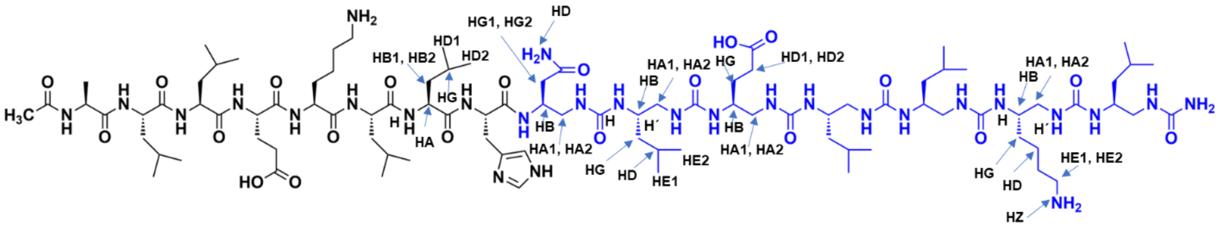

	H	H'	HA	HA1	HA2	HB	HB1	HB2	HD	HD1	HD2	HE	HE1	HE2	HG	HG1	HG2	HZ
A1	8.318	-	4.157	-	-	1.385	-	-	-	-	-	-	-	-	-	-	-	-
L2	8.255	-	4.145	-	-	-	1.588	1.666	-	0.8640	0.9100	-	-	-	-	-	-	-
L3	7.981	-	3.998	-	-	-	1.606	1.652	-	0.8500	0.8850	-	-	-	-	-	-	-
E4	7.885	-	3.940	-	-	2.070	-	-	-	-	-	-	-	-	-	2.315	2.370	-
K5	7.833	-	4.137	-	-	1.552	-	-	-	1.640	1.670	-	1.887	1.907	1.424	-	-	-
L6	8.050	-	4.028	-	-	-	1.608	1.748	-	-	-	-	-	-	-	-	-	-
L7	8.036	-	4.041	-	-	1.658	-	-	-	0.7530	0.7980	-	-	-	1.392	-	-	-
H8	8.079	-	4.483	-	-	3.245	-	-	-	-	-	-	-	-	-	-	-	-
Nu9	6.750	7.827	-	2.966	3.509	4.286	-	-	6.889	-	-	-	-	-	-	2.371	2.405	-
Lu10	5.861	5.955	-	2.450	3.373	3.773	-	-	-	-	-	0.7710	-	-	1.089	-	-	-
Eu11	5.718	6.211	-	2.684	3.505	3.684	-	-	-	1.527	1.749	-	-	-	-	2.280	2.349	-
Lu12	5.929	6.175	-	2.426	3.416	3.872	-	-	-	-	-	-	-	-	1.179	-	-	-
Lu13	6.107	6.402	-	2.761	3.280	3.746	-	-	-	-	-	0.8120	-	-	1.159	-	-	-
Ku14	5.892	-	-	2.532	3.516	3.705	-	-	-	-	-	-	-	-	-	-	-	4.454
Lu15	6.072	-	-	-	-	3.756	-	-	-	-	-	-	-	-	-	-	-	-

Table S3. ^{1}H NMR assignments of HPU-N

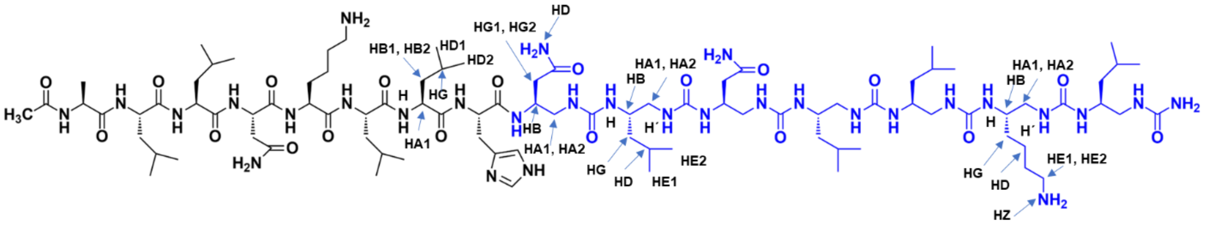

	H	H'	HA	HA1	HA2	HB	HB1	HB2	HD	HD1	HD2	HE	HE1	HE2	HG	HG1	HG2	HZ
Ac	-	-	-	-	-	1.938	-	-	-	-	-	-	-	-	-	-	-	-
A1	8.249	-	4.081	-	-	1.281	-	-	-	-	-	-	-	-	-	-	-	-
L2	8.196	-	4.145	-	-	-	1.508	1.558	-	0.7800	0.8240	-	-	-	-	-	-	-
L3	7.993	-	4.029	-	-	1.516	-	-	-	0.7660	0.8070	-	-	-	-	-	-	-
N4	8.051	-	4.398	-	-	2.710	-	-	-	-	-	-	-	-	-	-	-	-
K5	8.012	-	4.097	-	-	1.421	-	-	1.799	-	-	2.868	-	-	1.321	-	-	-
L6	8.050	-	4.055	-	-	1.476	1.641	-	-	0.7360	0.7820	-	-	-	1.643	-	-	-
L7	7.893	-	4.040	-	-	1.318	-	-	-	0.6940	0.7650	-	-	-	1.536	-	-	-
H8	8.011	-	4.408	-	-	-	3.085	3.178	7.239	-	7.240	-	-	-	-	-	-	-
Nu9	6.718	7.814	-	2.948	3.349	4.206	-	-	6.791	-	-	-	-	-	-	2.320	3.471	-
Lu10	5.840	5.957	-	2.526	3.246	3.679	-	-	-	-	-	-	0.6830	0.7170	1.055	-	-	-
Nu11	5.865	6.157	-	2.723	3.377	4.044	-	-	6.735	-	-	-	-	-	-	2.220	2.311	-
Lu12	5.766	6.090	-	2.572	3.253	3.785	-	-	1.526	-	-	0.7390	-	-	1.109	-	-	-
Lu13	5.978	6.260	-	2.748	3.176	3.669	-	-	1.504	-	-	0.7460	-	-	1.109	-	-	-
Ku14	6.349	-	-	2.558	-	3.627	-	-	-	1.339	1.404	1.564	1.543	1.556	-	1.224	1.274	2.874

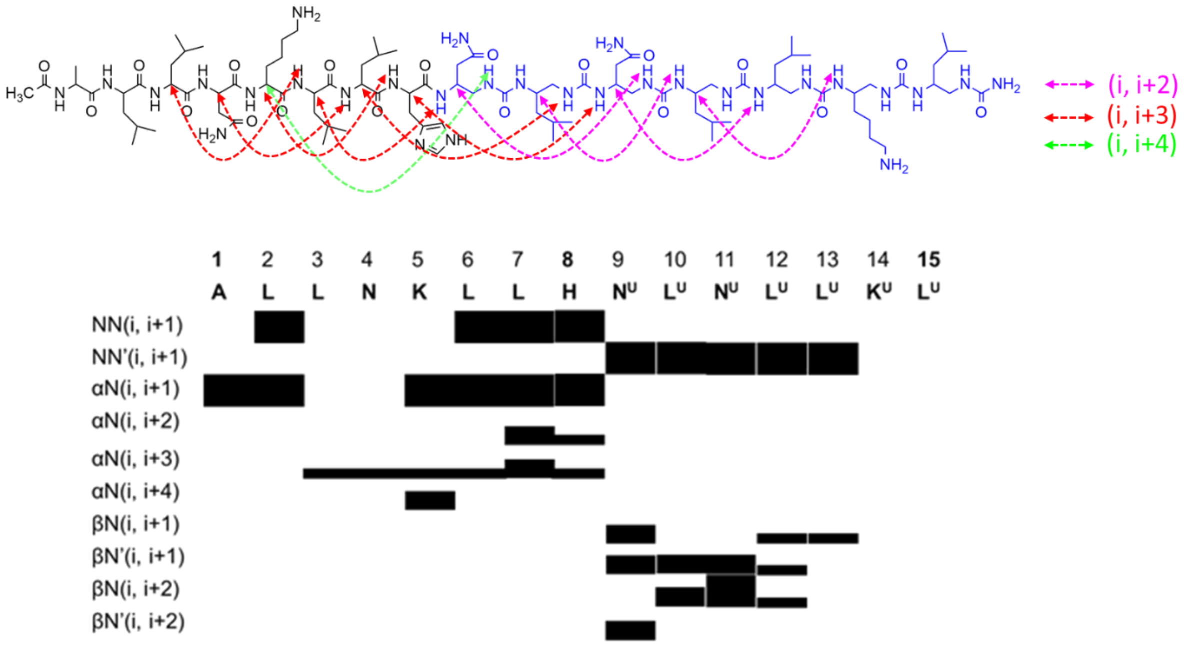

Figure S11. Cross peak NOEs of HPU-N obtained from NOESY spectrum. Medium-range and short-range NOEs of **HPU-N**. Schematic showing the intra residues contacts.

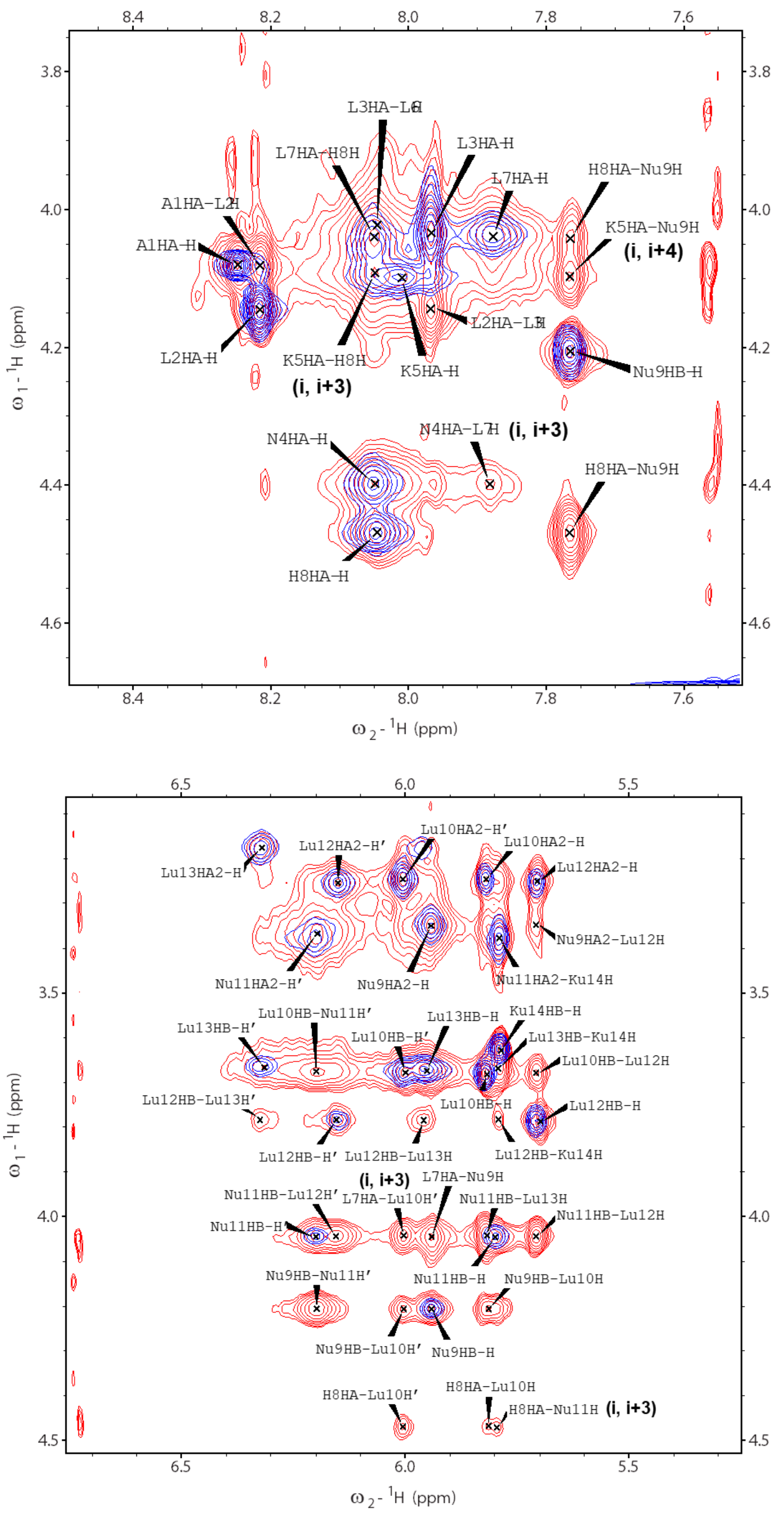

Figure S12. Partial TOCSY and NOESY spectra of HPU-N in H_2O at 298K. NOESY spectrum shows αN(i, i+1), αN(i, i+2), αN(i, i+3), αN(i, i+4) and β(i, i+1), β(i, i+2) cross peak NOEs. Blue and red indicate TOCSY and NOESY peaks, respectively.

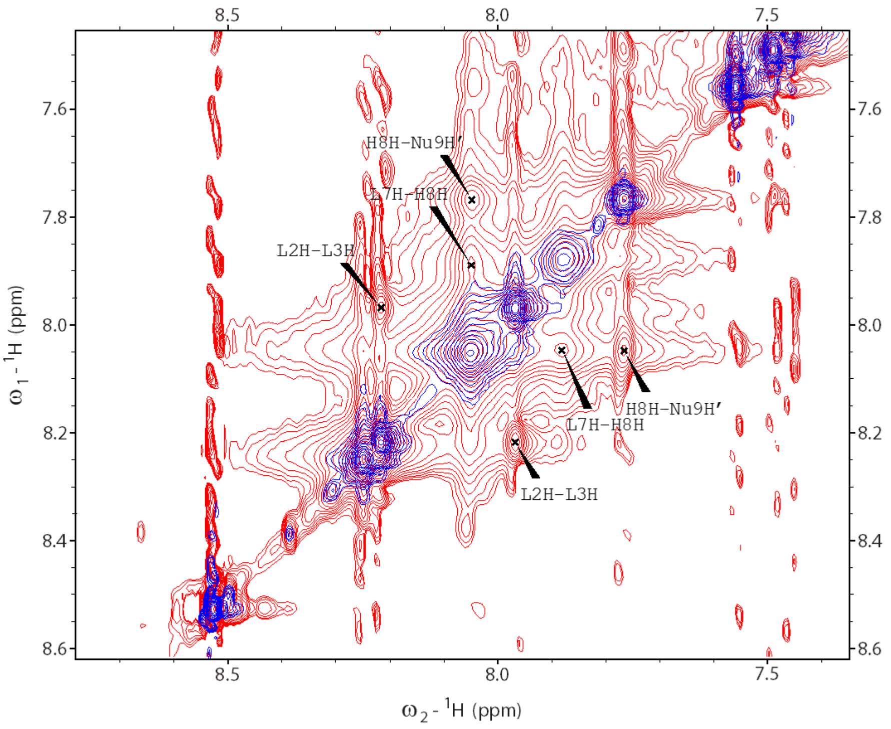

Figure S13. Partial TOCSY and NOESY spectra of HPU-N in H2O at 298K. NOESY spectrum shows the characteristic cross peak N,N'(i, i+1) NOEs of **HPU-N**. Blue and red indicate TOCSY and NOESY peaks, respectively.

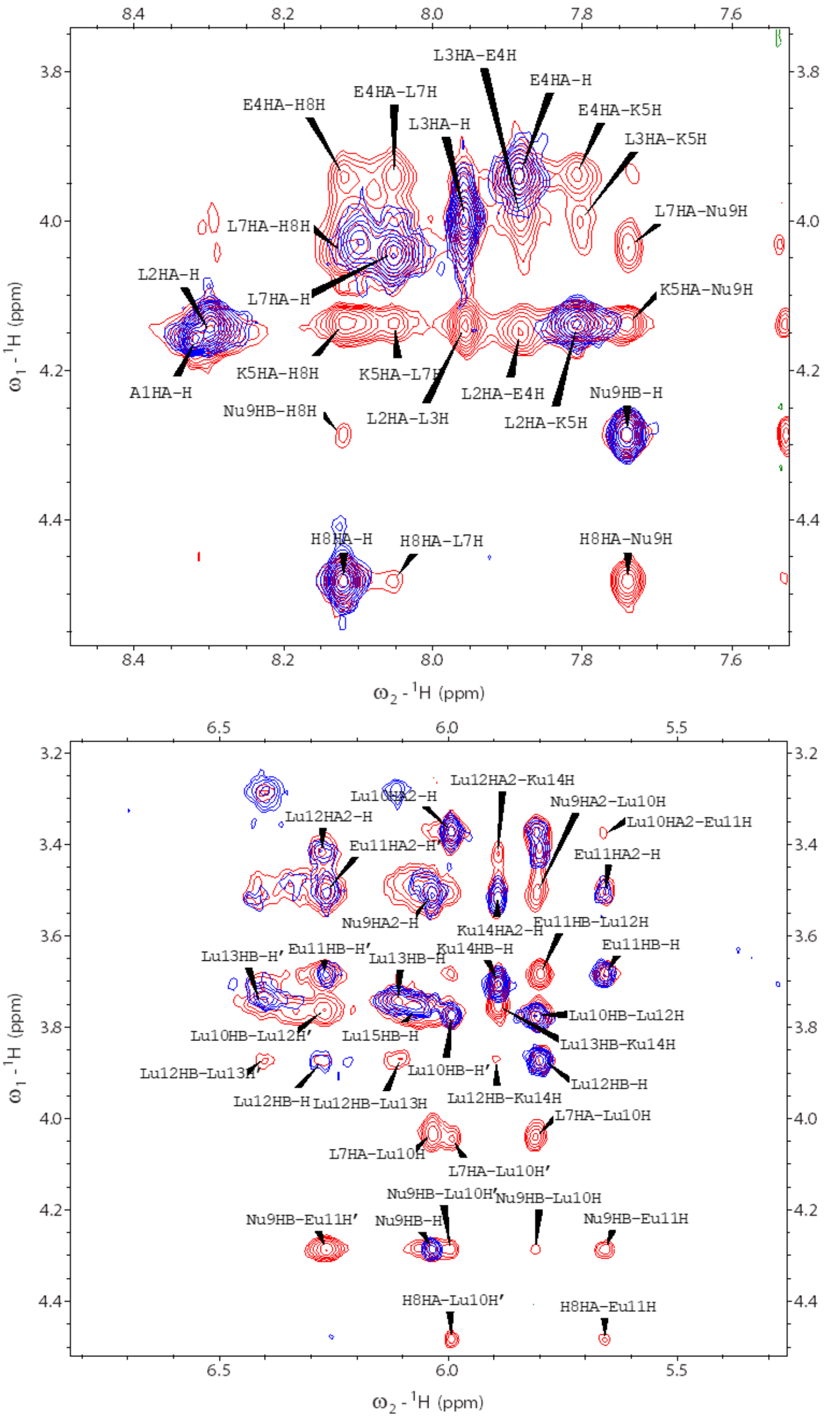

Figure S14. Partial NOESY spectra of HPU-E measured in H_2O at 298K. NOESY spectrum shows αN(i, i+1), αN(i, i+2), αN(i, i+3), αN(i, i+4) and β(i, i+1), β(i, i+2) cross peak NOEs. Blue and red indicate TOCSY and NOESY peaks, respectively.

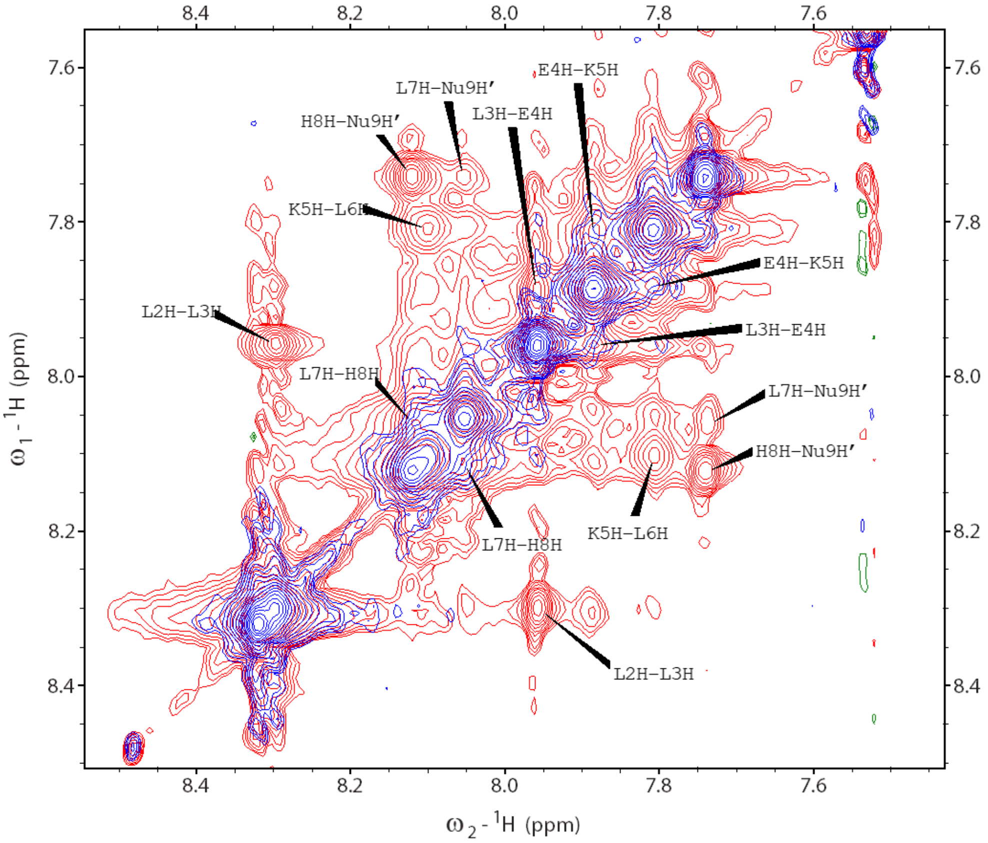

Figure S15. Partial NOESY spectra of HPU-E. It exhibits characteristic cross peaks N,N'(i, i+1) NOEs of **HPU-E** chimera in H_2O at 298K. Blue and red indicate TOCSY and NOESY peaks, respectively.

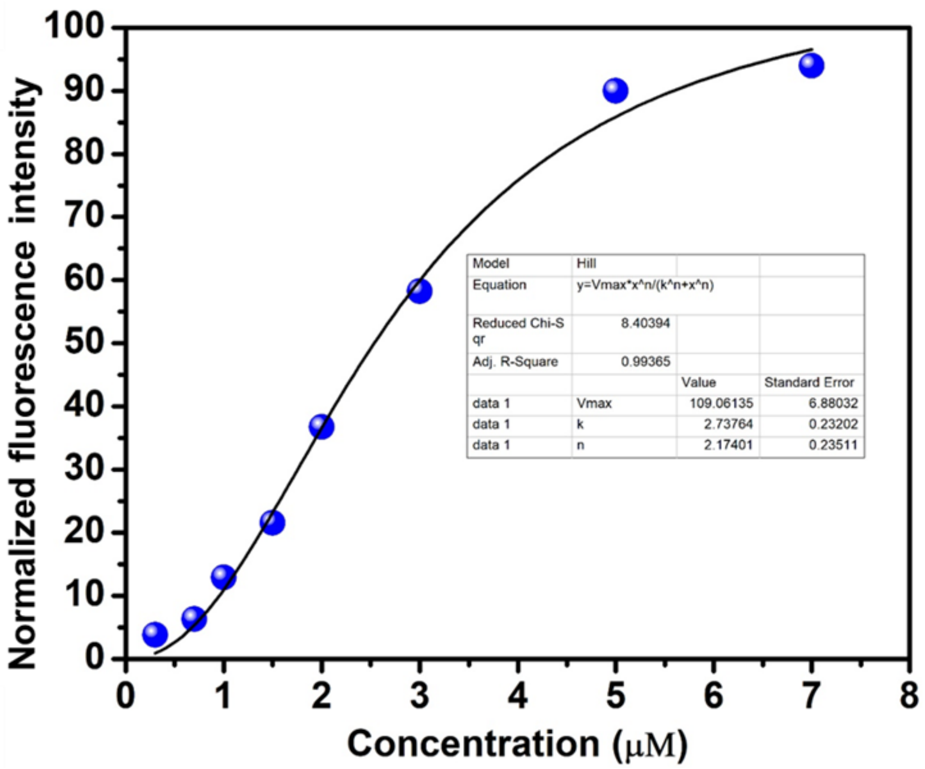

Figure S16. EC50 and Hill coefficient (n) of HPU-N was determined from the Hill analysis. The curve was plotted with normalized fluorescence intensity at 100 s against **HPU-N** concentration and determined EC50 and n to be 2.85±0.16 µM and 2.5±0.5 respectively, by fitting the curve using the Hill equation. Ion transport of **HPU-N** at 10 µM was avoided since it showed more than 100% fluorescence intensity. Maximum fluorescence intensity (100% here) was obtained after adding Triton X-100.

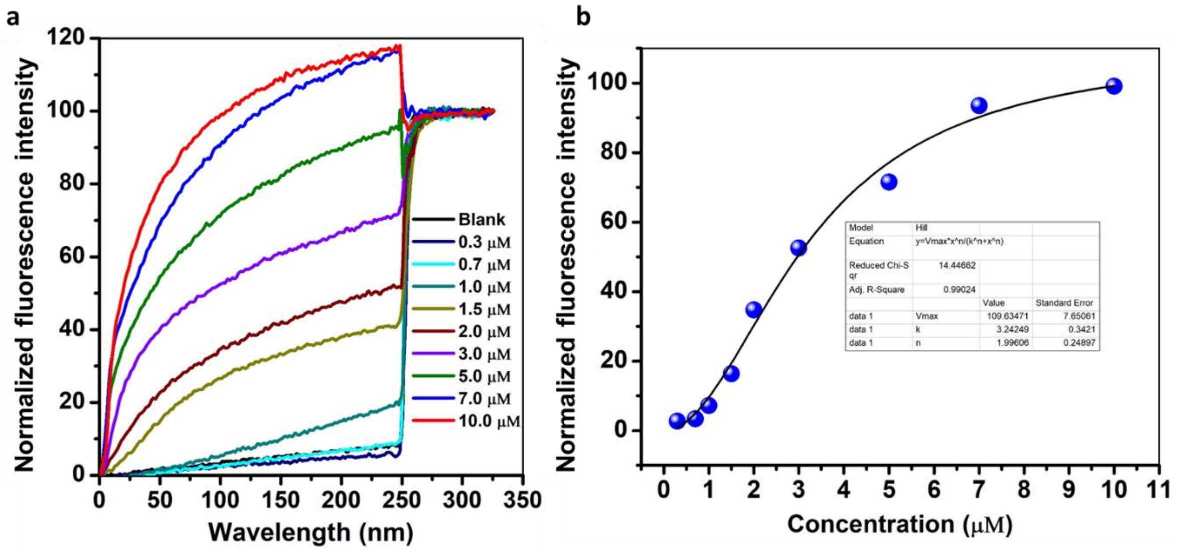

Figure S17. EC50 and Hill coefficient (n) of HPU-E was determined from the Hill analysis. a) Ion transport is represented as normalized emission intensity. b) The curve was plotted with normalized fluorescence intensity at 100 s against **HPU-E** concentration and observed EC50 and n to be 3.91±0.95 µM and 1.7 ±0.3 respectively, by fitting the curve using the Hill equation. Maximum fluorescence intensity (100% here) was obtained after adding Triton X-100.

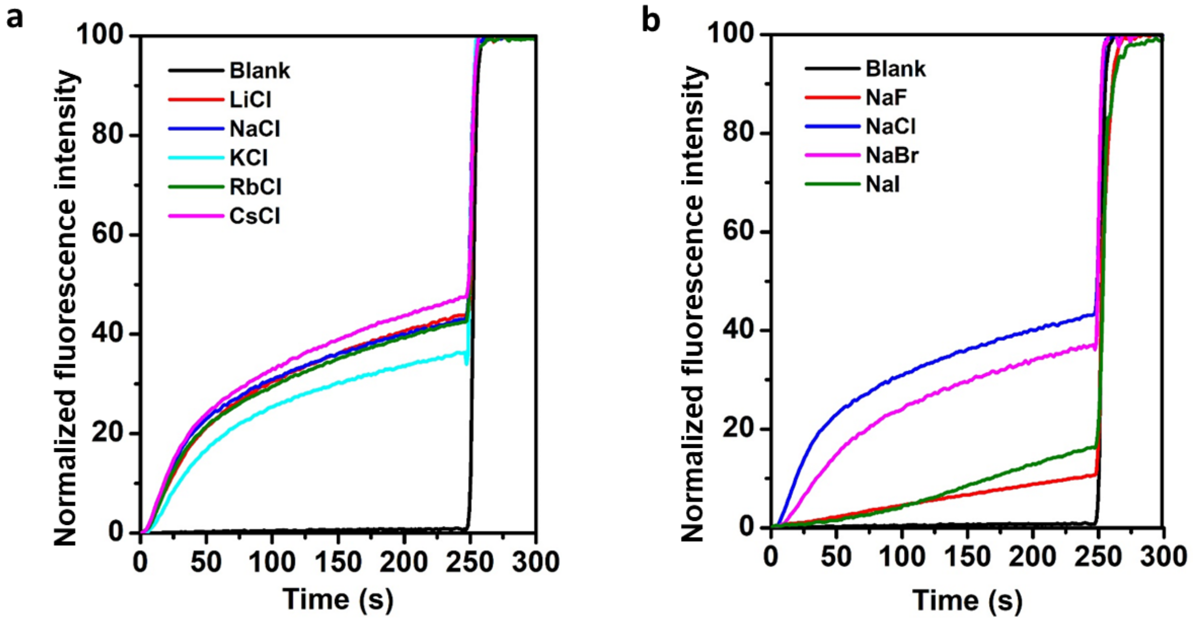

Figure S18. Ion selectivity of HPU-E. a) Identifying cation selectivity of **HPU-E** (2 µM) by HPTS assay after varying extravesicular cations MCl (where M^+=Li^+, Na^+, K^+, Rb^+, and Cs^+) with intravesicular NaCl. b) Identification of anion selectivity of **HPU-E** (2 µM) by HPTS assay after varying extravesicular anions NaX (where X^- = F^-, Cl^-, Br^-, I^-) with intravesicular NaCl.

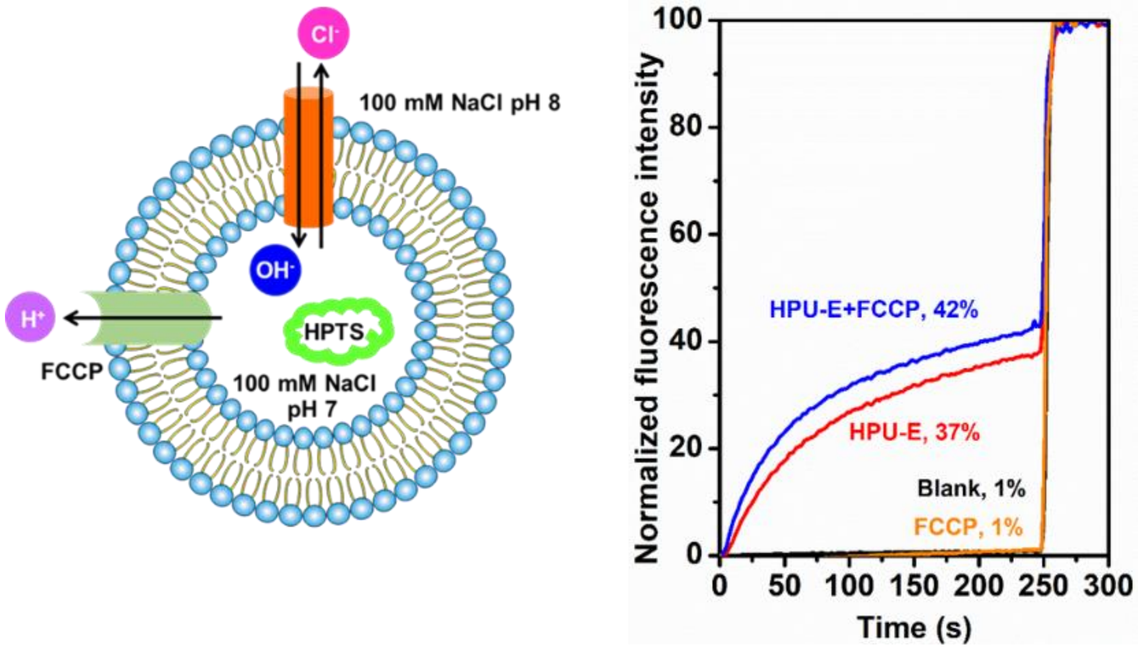

Figure S19. Comparison of ion transport activity of HPU-E with carbonyl cyanide-4-(trifluoromethoxy)-phenylhydrazone (FCCP). The ion transport activity of **HPU-E** (2 μM) in the presence and absence of proton transporter **FCCP** (2 μM) by HPTS assay.

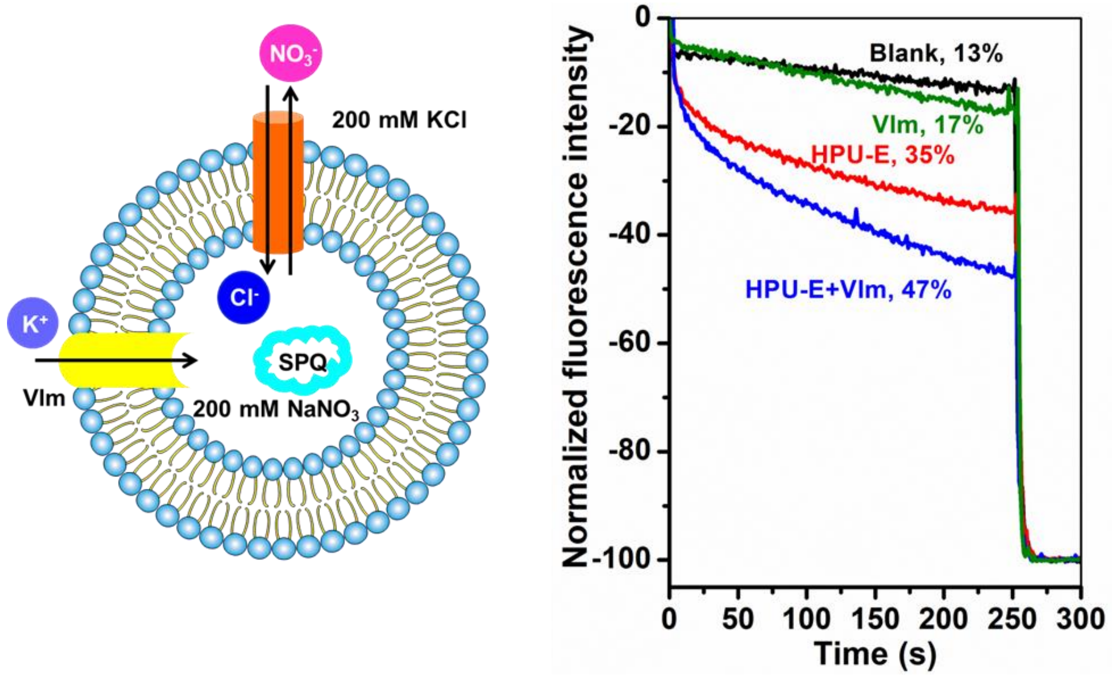

Figure S20. Comparison of ion transport activity of HPU-E with valinomycin (Vlm). Cl^- transport activity of **HPU-E** (2.5 μM) was measured in presence of potassium transporter valinomycin (100 nM) with intravesicular $NaNO_3$ and extravesicular KCl.

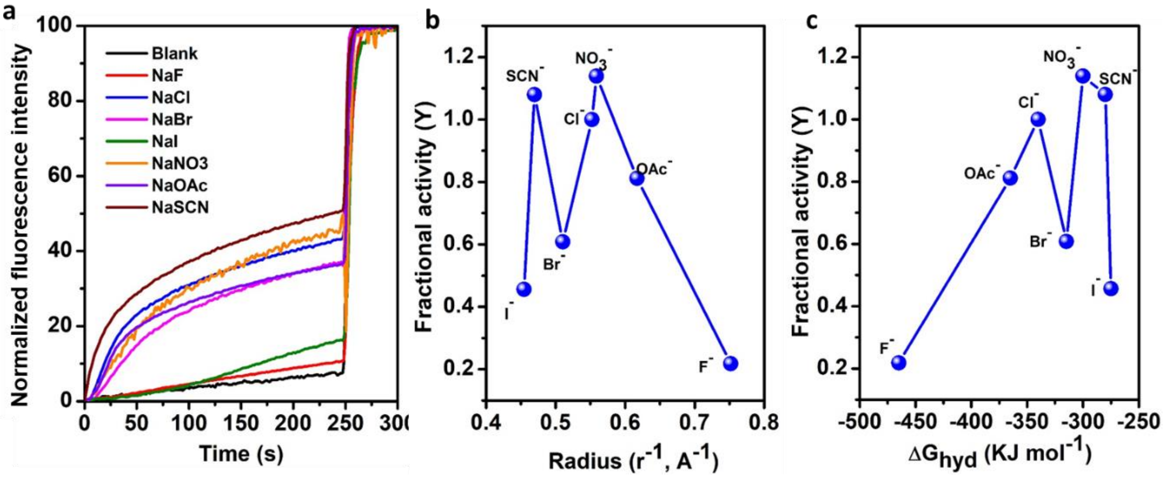

Figure S21. Anion selectivity is determined by fractional activity (Y). a) Anion selectivity trend of **HPU-E** determined by HPTS assay with varying extravesicular X^- ions (where X^- = F^-, Cl^-, Br^-, I^-, NO_3^-, OAc^-, SCN^-) with intravesicular Cl^- ion. Anion selectivity is depicted as normalized emission intensity. b, c), Fractional activity Y (relative to Cl^-) is represented here as a function of the reciprocal of anion radius (b) and hydration energy (c).

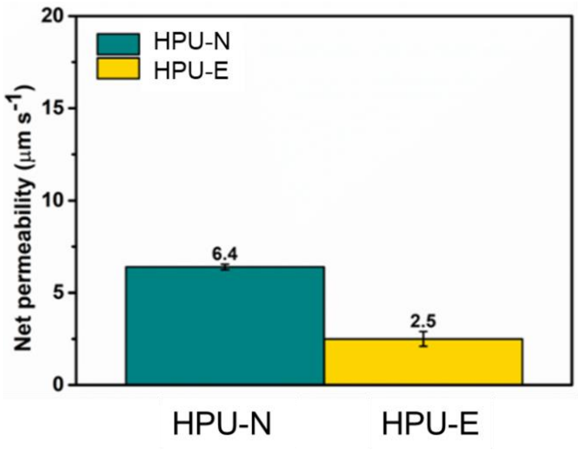

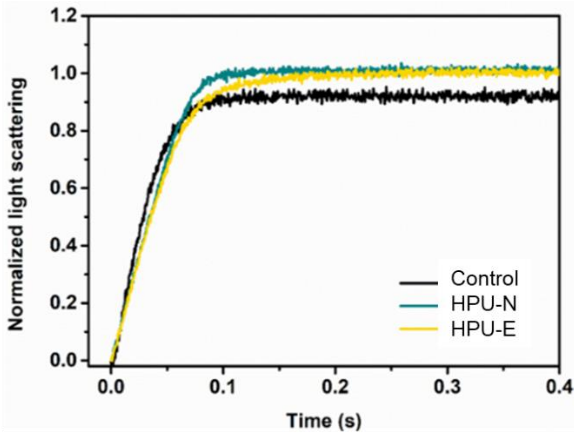

Figure S22. Water permeability of HPU-N and HPU-E. The stopped-flow traces of **HPU-N** and **HPU-E** at 5 μM with sucrose as osmolyte (Δosm=600 mOsmol/kg) showing water permeability.

Calculation of number of channels from cryo-EM images

According to the results of 2D average, the average distance between each two channels is 3.7 nm, so each channel occupies an area of 3.7 nm x 3.7 nm, the number of channels per unit area is:

$$\frac{1}{(3.7\,nm \times 3.7\,nm)} = \frac{1\times10^6}{(3.7\,\mu m \times 3.7\,\mu m)} = 73046/\mu m^2$$

The radius of the imaged liposome was 43.6 nm. The total surface area of liposome was $4\pi r^2$ = 23930.85 nm^2. Therefore, total number of channels per liposome = no. of channels x surface area of liposome = 1748 channels per liposome at 1:100 molar ratio.

Single-channel water permeability calculation

Single-channel water permeability was calculated based on the number of **HPU-N** channels inserted in PC/PS (4:1) liposomes as determined by cryo-EM imaging. As mentioned above, the calculated channel number was 1748 per liposome at 1:100 molar ratio. The radius of **HPU-N**-embedded liposomes for the stopped-flow assays was 50.0 nm. The sum of outer and inner surface areas of lipid is S(outer + inner) = $4\pi r^2 + 4\pi(r - 5.6)^2$ = 58073.42 nm2, r = 50.8 nm, the bilayer thickness was 5.6 nm (obtained from cryo-EM studies).

S(average) = S(outer + inner)/2 = 29036.71 nm^2

Number of **HPU-N** channels per vesicles = 1748

The overall net permeability of **HPU-N** channels in liposomes was **P0** = 6.4 μm/s

The single-channel permeability (**P1** cm^3/s) was calculated as: 6.4 × S(average)/Nc = (6.4 × 29036.71 × 10^{-18})/1748 cm^3/s = 1.06 × 10^{-16} cm^3/s

The single-channel permeability (**P2** water molecules/s) was calculated as: **P1** × 6.022 × $10^{23}/18 = (1.06 \times 10^{-16} \times 6.022 \times 10^{23})/18 = 3.5 \times 10^{6}$ H_2O/s

P1$_{\mathbf{HPU\text{-}N}}$ **= 1.06 × 10^{-16} cm^3/s**

P2$_{\mathbf{HPU\text{-}N}}$ **= 3.5 × 10^{6} water molecules/s**

Owing to the sequence similarity of HPU-N and HPU-E, we assumed same number channel formation.

Number of HPU-E channels per vesicles = 1748. The overall net permeability of **HPU-E** channels in liposomes was **P0** = 2.5 µm/s

The single-channel permeability (**P1** cm^3/s) was calculated as: 2.5 × S(average)/Nc = (2.5 × 29036.71 × 10^{-18})/1748 cm^3/s = 0.41 × 10^{-16} cm^3/s

The single-channel permeability (**P2** water molecules/s) was calculated as: **P1** × 6.022 × $10^{23}/18 = (0.41 \times 10^{-16} \times 6.022 \times 10^{23})/18 = 1.3 \times 10^{5}$ H_2O/s

P1$_{\mathbf{HPU\text{-}E}}$ **= 0.40 × 10^{-16} cm^3/s**

P2$_{\mathbf{HPU\text{-}E}}$ **= 1.3 × 10^{5} water molecules/s**

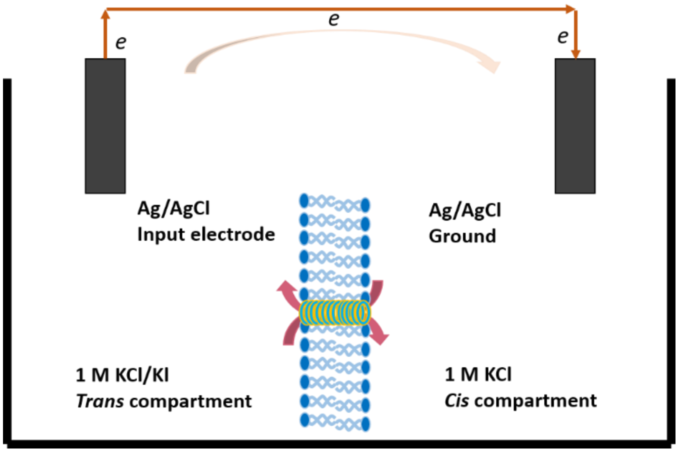

Scheme S2. Schematic illustration of the patch-clamp technique for recording single channel currents.

Table S4. Data collection and refinement statistics for the X-ray crystal structure of HPU-N.

Crystallization condition	0.1 M MES pH 6.2,<br>0.6 M KCl
Data collection	
Space group	*I* 4 3 2
a, b, c (Å)	65.19, 65.19, 65.19
α, β, γ (°)	90.00, 90.00, 90.00
Resolution (Å)	46.09 – 1.77 (1.84 – 1.76)
R_{meas} (%)	5.2 (211)
I / σ	47.6 (2.6)
Reflections (total)	184089
Reflections (unique)	2539
Completeness (%)	100 (100)
Redundancy	72.5 (66.2)
Refinement	
Resolution (Å)	46.09 – 1.77
R_{work} / R_{free} (%)	25.35 / 27.16
Atoms	148
Waters	9
Overall B-factor (Å^2)	50.14
R.m.s. deviations	
Bond-lengths (Å)	0.015
Bond-angles (°)	2.685
CCDC code	2222283

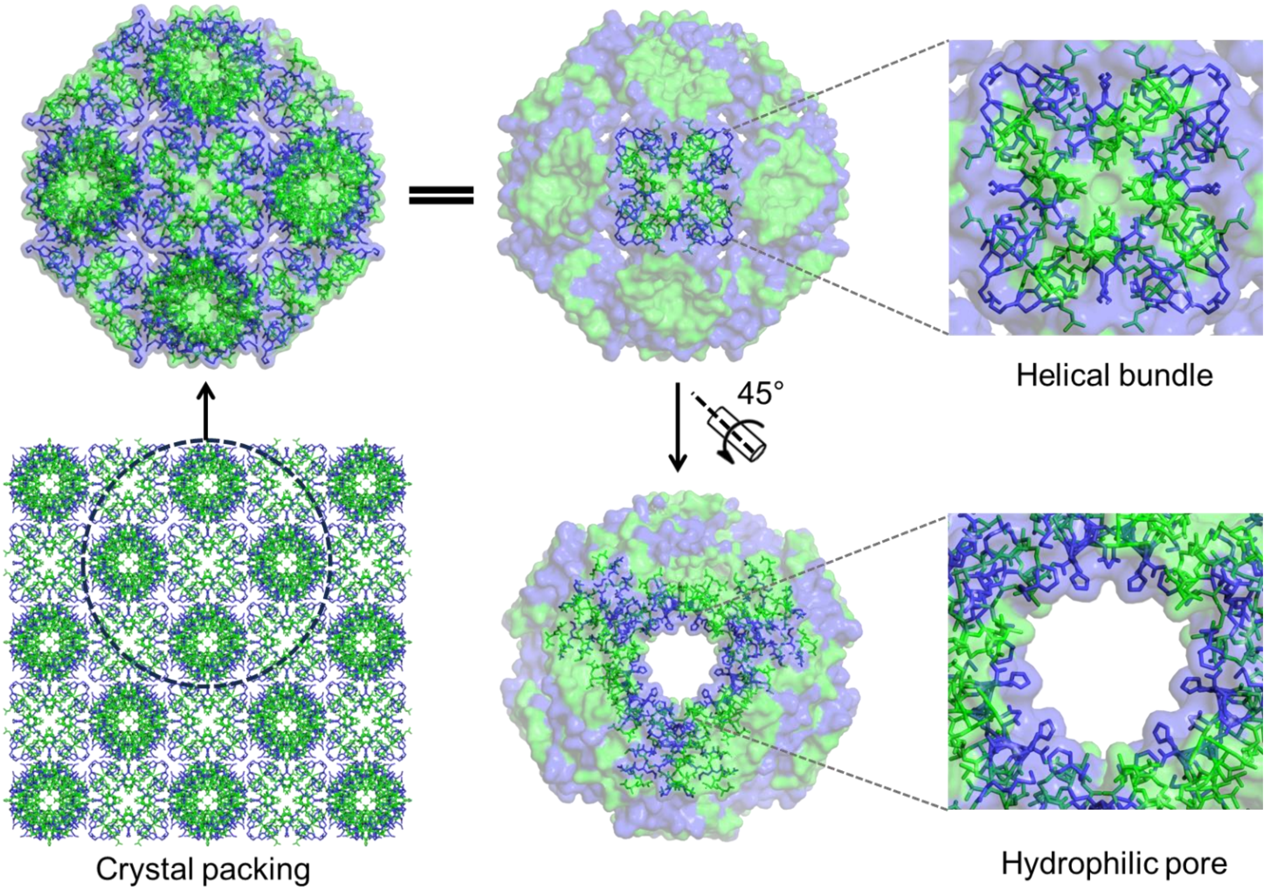

Figure S23. Crystal packing of HPU-N. It shows the presence of hydrophobic bundles and a hydrophilic pore. Polar and hydrophobic residues marked as blue and green respectively

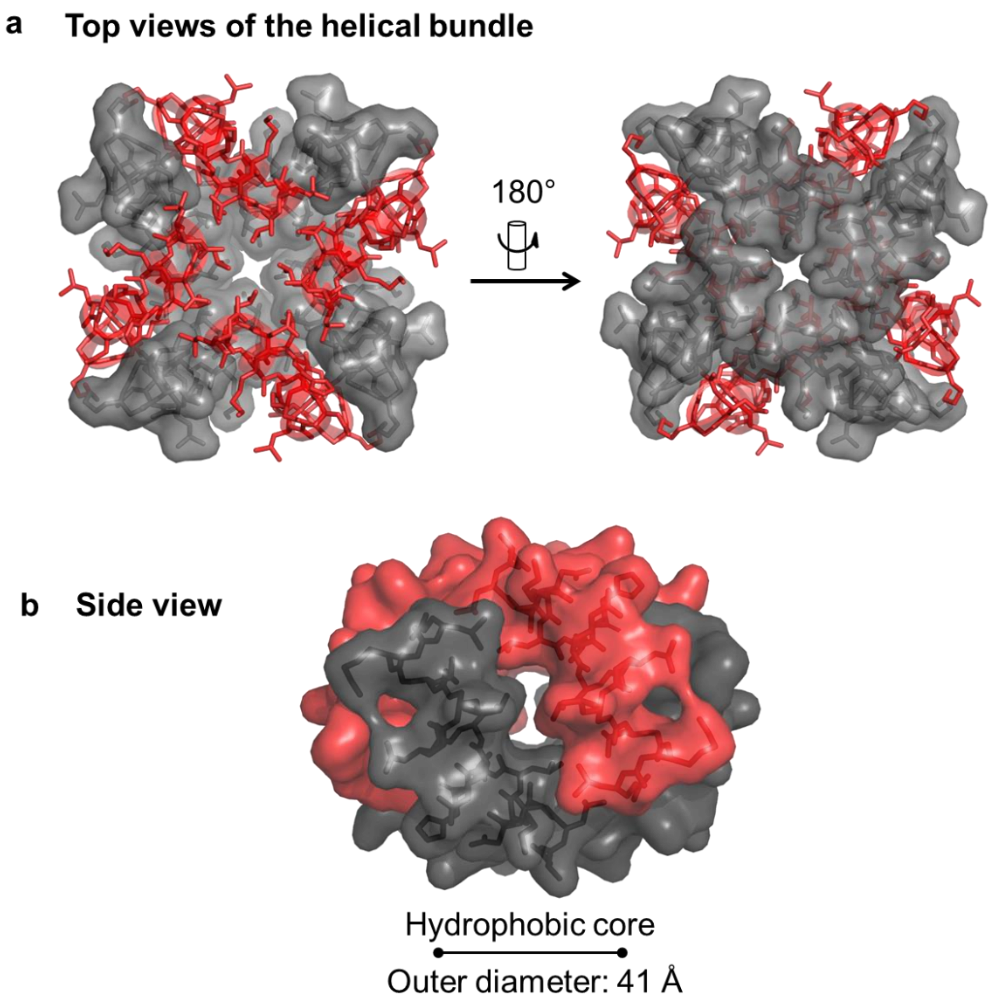

Figure S24. Crystal structure shows the helical bundle of HPU-N. (A) Top views and (B) side view of helix dimer repeat unit of the helix bundle displays each side of the bundle in two different colours in stick, cartoon, and surface model. In the octameric helical bundle arrangement two chimera helices pack in an anti-parallel way forming a cavity.

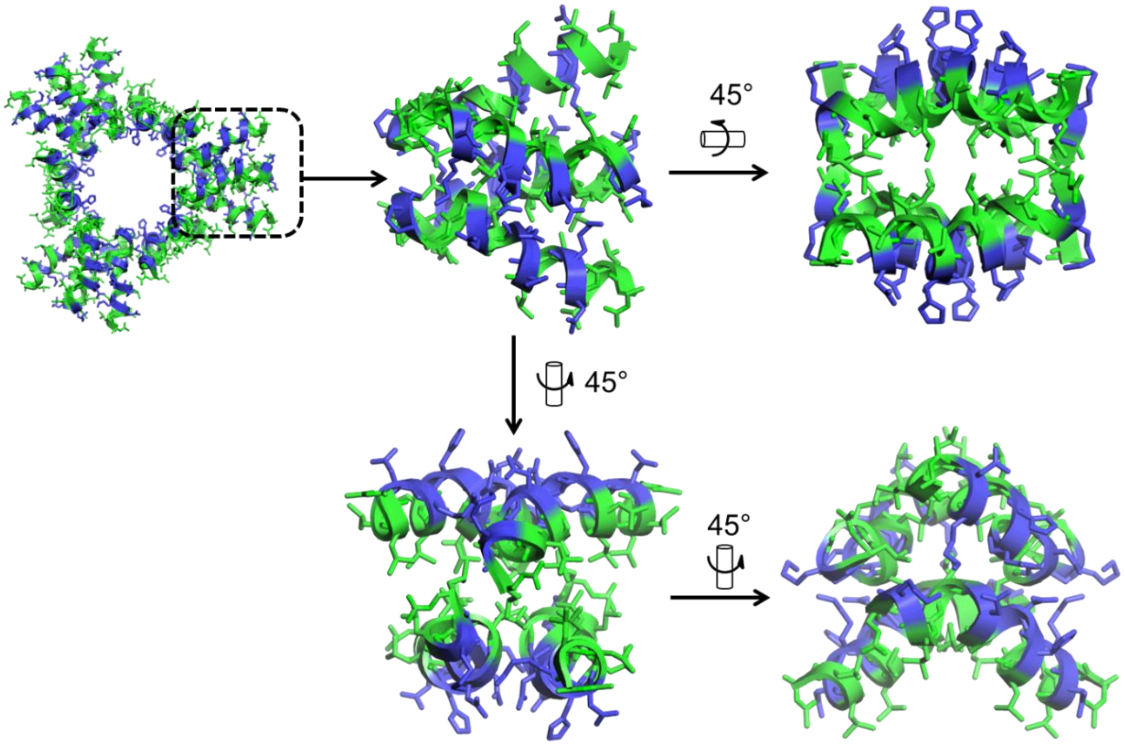

Figure S25. Crystal packing of HPU-N shows Hydrophobic interaction. Inter-helix packing in hydrophilic pore is stabilized by hydrophobic Leu^{U}-Leu interaction showing in different views. Polar and hydrophobic residues marked as blue and green respectively.

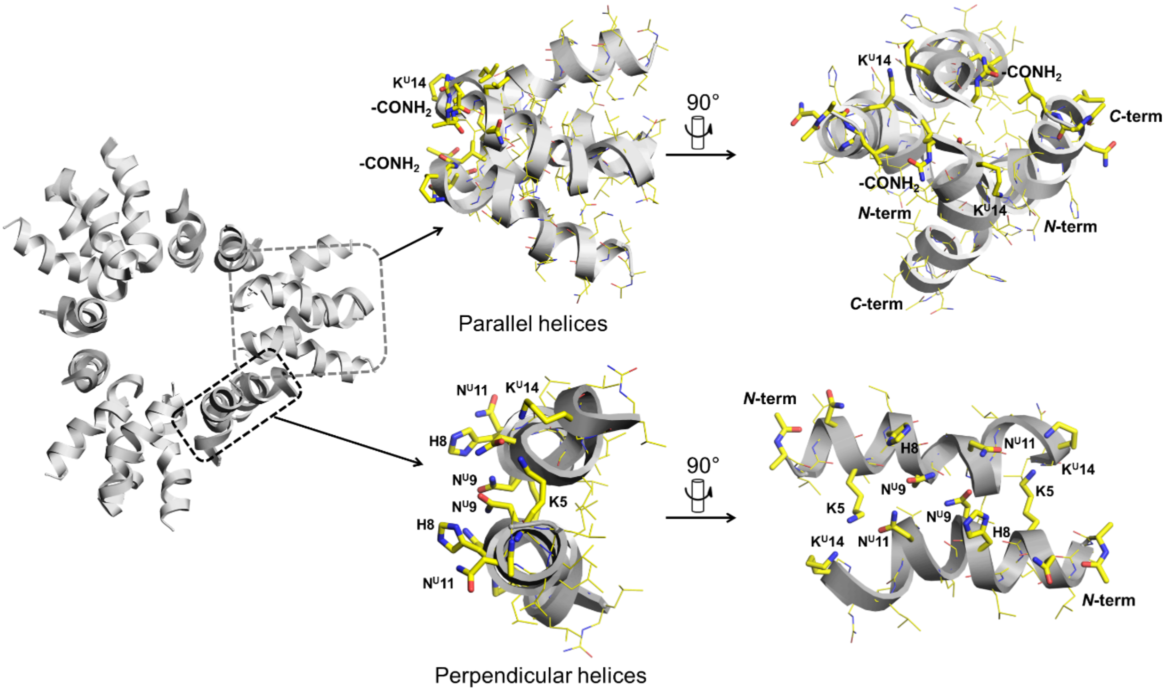

Figure S26. Pore residues in hydrophilic pore of HPU-N. Crystal figures shows the arrangement of helices within the hydrophilic pore, with a particular focus on highlighting the presence of polar residues in the vicinity of the pore region.

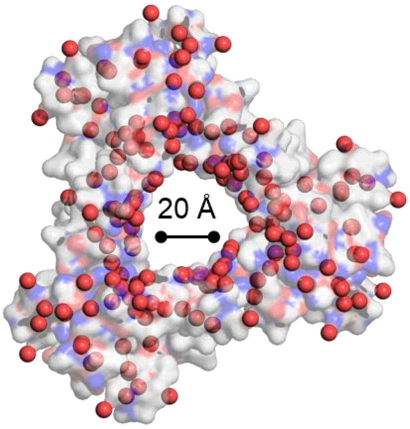

Figure S27. Crystal structure shows the water molecules in the Hydrophilic pore of HPU-E. The surface model shows the hydrophilic pore associated with water molecules. Red spheres represent water molecules. Water molecules bind to the polar residues such as Asn, Asn^U and Lys, Lys^U by an intricate H-bonding pattern.

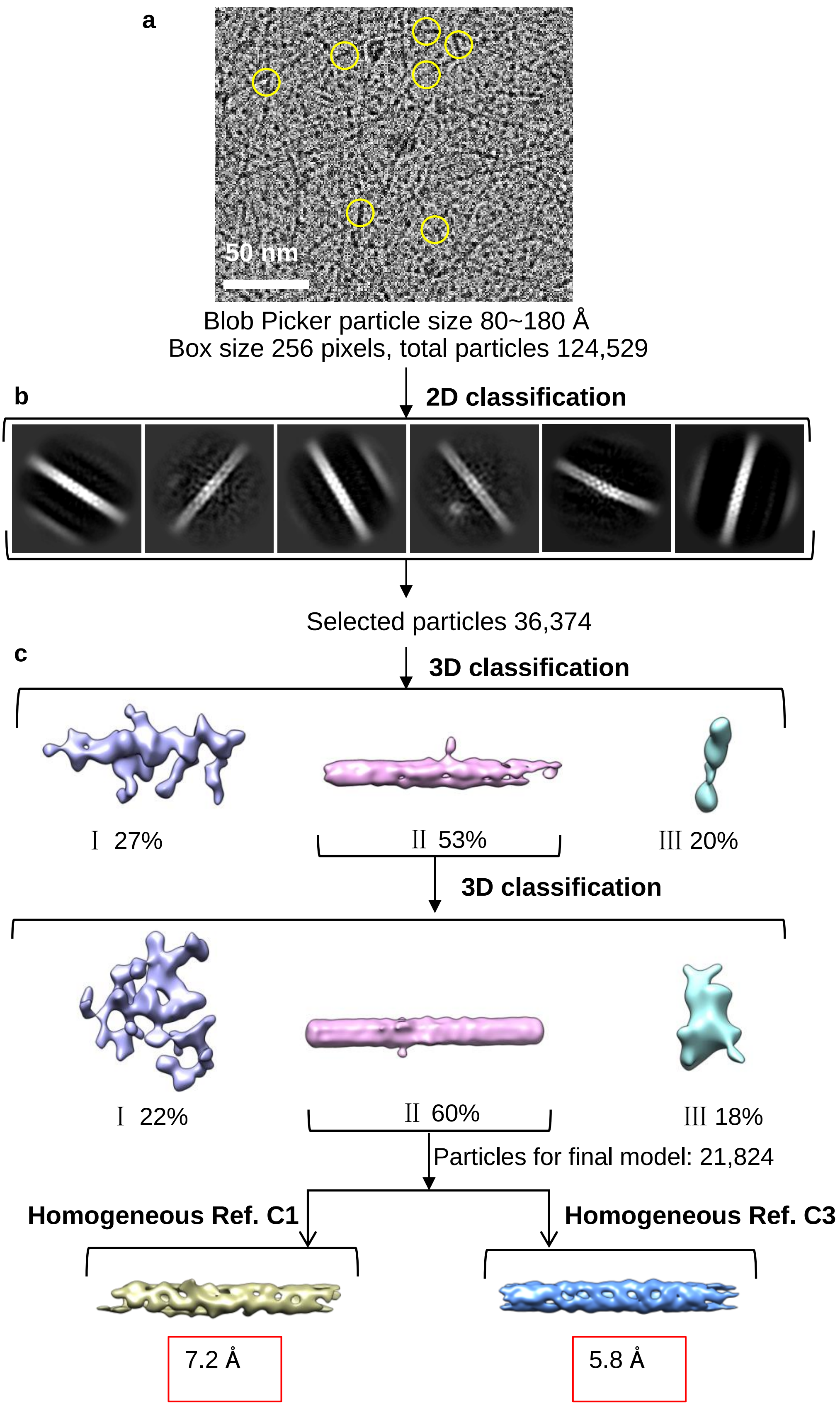

a
50 nm
Blob Picker particle size 80~180 Å
Box size 256 pixels, total particles 124,529
b
2D classification
Selected particles 36,374
c
3D classification
Ⅰ 27%
Ⅱ 53%
Ⅲ 20%
3D classification
Ⅰ 22%
Ⅱ 60%
Ⅲ 18%
Particles for final model: 21,824
Homogeneous Ref. C1
Homogeneous Ref. C3
7.2 Å
5.8 Å

Figure S28. Single-particle cryo-electron microscopy investigation of the HPU-N chimera. (A) Representative depiction of a cryo-EM micrograph capturing the **HPU-N** chimera. (B) Two-dimensional ensemble averages of the cryo-EM particle micrographs, each encompassing bounding box dimensions spanning 270 Å. (C) Flowchart illustrating the sequential progression of image processing procedures. The conclusive density maps, characterized by their respective global resolutions, are demarcated within red-bordered compartments. Specifically, the density map exhibiting C3 symmetry is designated for subsequent rigorous structural analysis.

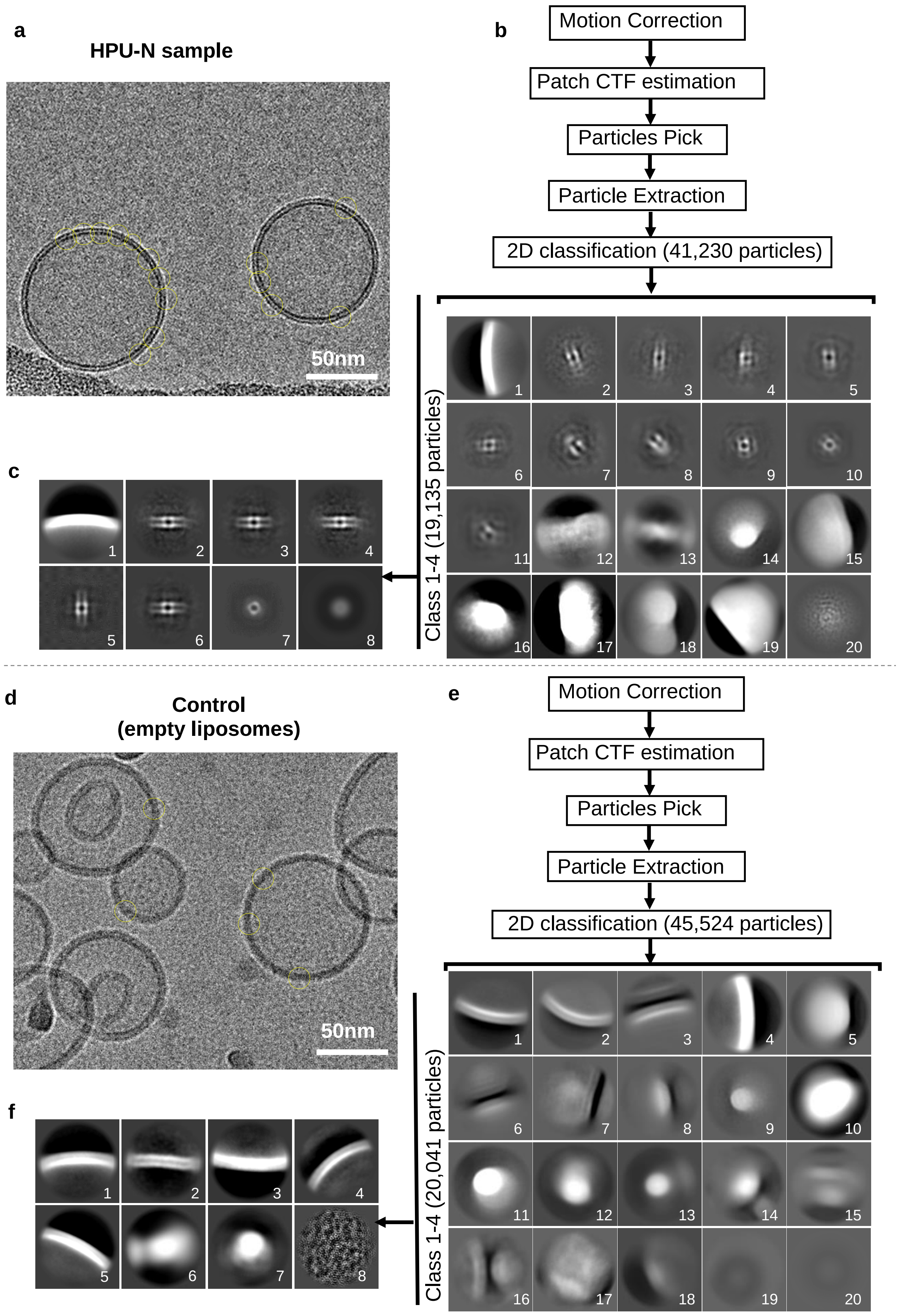
a
HPU-N sample
50nm
b
Motion Correction
Patch CTF estimation
Particles Pick
Particle Extraction
2D classification (41,230 particles)
Class 1-4 (19,135 particles)
c
d
Control
(empty liposomes)
50nm
e
Motion Correction
Patch CTF estimation
Particles Pick
Particle Extraction
2D classification (45,524 particles)
Class 1-4 (20,041 particles)
f

Figure S29. Flowchart of 2D average image processing of HPU-N in liposomes. (A) Raw micrograph of **HPU-N** in liposome cryo-EM sample with particle picker box. (B) Sequential processing flowchart detailing the steps employed for generating 2D average images of **HPU-N** in liposomes. (C) Last round of 2D average of **HPU-N** sample images. (D) Raw micrograph of the cryo-EM specimen featuring control liposomes, accompanied by the marked particle picker box. (E) Flowchart outlining the iterative procedure for generating 2D average images of the control liposomes. (F) Last round of 2D average of control liposome images.

References (Supplementary)

[1] C. Douat-Casassus, K. Pulka, P. Claudon, G. Guichard, *Org. Lett.* **2012**, *14*, 3130.

[2] S. H. Yoo, B. Li, C. Dolain, M. Pasco, G. Guichard, *Methods Enzymol.* **2021**, *656*, 59.

[3] G. W. Collie, K. Pulka-Ziach, C. M. Lombardo, J. Fremaux, F. Rosu, M. Decossas, L. Mauran, O. Lambert, V. Gabelica, C. D. Mackereth, G. Guichard, *Nat. Chem.* **2015**, *7*, 871.
